# Mass Spectrometric Fingerprint Mapping Reveals Species-Specific Differences in Plant Polyphenols and Related Bioactivities

**DOI:** 10.3390/molecules28176388

**Published:** 2023-08-31

**Authors:** Suvi Vanhakylä, Juha-Pekka Salminen

**Affiliations:** Natural Chemistry Research Group, Department of Chemistry, University of Turku, FI-20014 Turku, Finland; smhvan@utu.fi

**Keywords:** bioactivity, chemical diversity, chemotaxonomy, flavonols, oxidative activity, phytochemistry, protein precipitation, tannins

## Abstract

Plant species show large variation in the composition and content of their tannins and other polyphenols. These large metabolites are not easy to measure accurately, but they are important factors for species bioactivity and chemotaxonomy. Here, we used an automated group-specific UHPLC-DAD-MS/MS tool to detect and quantify eight most common polyphenol groups in 31 chemically diverse plant species representing many types of growth forms and evolutionary ages. Ten replicate plants were used for each species and two polyphenol-related bioactivities, i.e., protein precipitation capacity and oxidative activity were measured in all samples as well. By the help of a novel 2D fingerprint mapping tool we were able to visualize the qualitative and quantitative differences between the species in hydrolysable tannins (galloyl and hexahydroxydiphenoyl derivatives), proanthocyanidins (procyanidins and prodelphinidins), flavonols (kaempferol, quercetin and myricetin derivatives) and quinic acid derivatives together with the two bioactivities. The highest oxidative activities were found with species containing ellagitannins (e.g., *Quercus robur*, *Geranium sylvaticum*, *Lythrum salicaria* and *Chamaenerion angustifolium*) or prodelphinidin-rich proanthocyanidins (e.g., *Ribes alpinum*, *Salix phylicifolia* and *Lysimachia vulgaris*). The best species with high protein precipitation capacity were rich in gallotannins (*Acer platanoides* and *Paeonia lactiflora*) or oligomeric ellagitannins (e.g., *Comarum palustre*, *Lythrum salicaria* and *Chamaenerion angustifolium*). These types of tools could prove their use in many types of screening experiments and might reveal even unusually active polyphenol types directly from the crude plant extracts.

## 1. Introduction

Plants produce a huge number of specialized metabolites for their own protection; several thousands of compounds can be detected from a single species using modern high-resolution mass spectrometric (HR-MS) tools when the compounds are first at least partially separated by ultrahigh performance liquid chromatography (UHPLC) or other efficient separation techniques [1]. These approaches are very useful in revealing especially the metabolomic pool of small-molecular-weight compounds in plants, but larger compounds might not be detected at all depending on the specific parameters optimized for sample preparation, mass spectrometric ionization and ion selection and detection. Such a pool of thousands of smaller metabolites detected per plant species forms a great chemotaxonomic resource, since a carefully selected set of these individual compounds can function as chemotaxonomic markers effectively discriminating many of the species [1,2,3,4,5]. What makes this approach a very efficient one is the fact that the identity of the detected ions and the corresponding compounds do not have to be known. Given the numbers of ions detected per species, good knowledge of most of the compounds cannot be even expected, although the databases and algorithms for compound annotation and identification on the basis of their chromatographic and mass spectrometric features do help in this tedious task [6,7,8,9,10].

Another good option for discriminating plant species on the basis of their chemotaxonomic features is to select known compounds—perhaps even the major metabolites of species—for specific detection using a HR-MS or multiple reaction monitoring (MRM) tools by a triple quadrupole MS equipment. The benefit of knowing what is detected in each species can be utilized in conclusions that are drawn regarding the exact biosynthetic linkages or evolutionary origins between the compounds, or even of their significance to the bioactivities observed for the species. The obvious shortcoming of this approach is that it will not detect the whole pool of compounds responsible for a specific biosynthetic branch or bioactivity type. Thus, the selection of the most proper marker compounds, e.g., per each biosynthetic branch is crucial, if the existence of these branches in specific species is to be determined.

**Figure 1:**
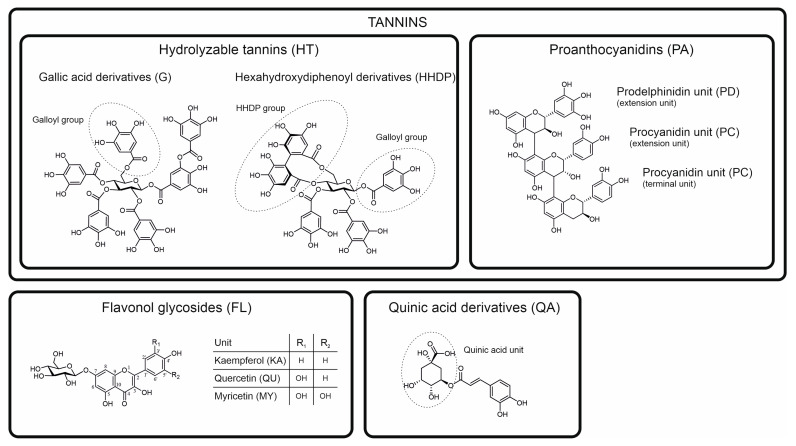
Classification and examples of model structures of the main polyphenol groups and their subgroups detected and quantified in this study by the group-specific multiple reaction monitoring methods developed by Engström et al. [11,12]. Hexagalloylglucose (**top left**) is an example of pure galloyl derivatives and tellimagrandin II (**top middle**) of ellagitannins containing hexahydroxydiphenoyl groups and in some cases also galloyl groups. The trimeric proanthocyanidin (**top right**) contains two procyanidin and one prodelphinidin unit. The three flavonol derivative groups (**bottom left**) detected in the water-soluble extracts of this study are almost invariably glycosides, with the example showing a 7-*O*-glucoside. The quinic acid derivative (**bottom right**) is a 5-*O*-caffeoyl quinic acid.

In this paper, we aimed to test our own approach for chemotaxonomic separation of species by utilizing the benefits of both of the approaches described above. We chose poly-phenols as one of the most common groups of specialized metabolites responsible for producing marker compounds widely detectable in the plant kingdom. Of these, we selected the four most common subgroups of tannins and three most common subgroups of flavonoids together with common quinic acid derivatives as the ubiquitous sources of chemotaxonomic markers (Figure 1). Instead of doing the classical targeted MRM detection of individual marker compounds within each of these polyphenol groups, we focused on the targeted detection using MRM of functional units present in all compounds within these groups [11,12]. This approach is thus targeted for the compound groups containing those specific functional units, but untargeted for the compounds per se. The functional units are first fragmented from the compounds in the ion source, and then, the fragments are subjected to their specific MRM detection. For instance, one such group-specific MRM method detects all ellagitannins that contain one or more hexahydroxydiphenoyl (HHDP) units. With these types of MS/MS tools, [11,12] we aimed to detect all individual compounds in the galloyl (G) and HHDP derivatives (hydrolysable tannins (HT)), procyanidin (PC) and prodelphinidin (PD) derivatives (proanthocyanidins (PA), syn. condensed tannins), kaempferol (KA), quercetin (QU) and myricetin (MY) derivatives (flavonols (FL)) and quinic acid (QA) derivatives (e.g., caffeoyl, coumaroyl and galloyl quinic acids). In practice, this approach combines both targeted (compound groups) and non-targeted (compounds) analytics and results into a single 2D fingerprint per each compound group [13] (see also Figure S1–S31) that can be converted to mg/g dry weight quantitation.

To make the included polyphenol group quantifications even more meaningful, we complemented our approach by the measurement of two polyphenol-related bioactivities: the protein precipitation capacity (PPC) and oxidative activity (OX). This way we could aim to link the observed bioactivity levels in plants to their polyphenols that have known PPC and/or OX, or to find polyphenols that are yet unknown for these bioactivity types. Protein precipitation capacity is very traditional bioactivity measure for tannins; especially for hydrolysable tannins, there is a good structure–activity knowledge available [14,15]. The same is true for oxidative activity [16,17] that can be triggered by, e.g., metal ions, high pH or plant enzymes [18,19]. Here, we focused on the alkaline oxidative activity, since plants defend themselves against insect herbivores, some of which have alkaline gut pH [20]. Oxidation of polyphenols at high pH in the presence of oxygen can yield quinones and hydrogen peroxide. Quinones as such can be harmful as they bind with the protein amino acids, and hydrogen peroxide can oxidize Fe^2+^ into Fe^3+^, thus producing a hydroxyl radical as well. In the presence of powerful antioxidants such as vitamin C, Fe^3+^ is reduced back to Fe^2+^ and this redox cycle, the so-called Fenton reaction, can form a constant source of hydroxyl radicals as long as polyphenols are oxidized in the presence of oxygen. This way some of the polyphenols can transform at high pH into harmful compounds with oxidative activity, while other polyphenols are not oxidized at high pH at all [19]. This way the oxidative activity can rank plants and their polyphenols by their ease of oxidation at these specific conditions. In other types of conditions that do not favor the production of quinones and other harmful side products of oxidation, these same polyphenols could in fact be powerful antioxidants and thus beneficial instead of harmful.

To test the functioning of our new approach, we selected a diverse set of 31 plant species and 10 species-specific replicate plants for this experiment. Species were selected by three criteria: (1) they should contain a highly diverse polyphenol content to test their effects on low vs. high bioactivities [19], (2) they should contain different growth forms including woody and herbaceous species, dwarf shrubs and evergreen and deciduous species to resolve growth form’s effect on the scale of the variance within a species, and (3) the species set should contain both phylogenetically distant and closely related species to find patterns in their polyphenol and/or bioactivity profiles that could be linked to their phylogenetic distance. A subset of 10 tree species was selected so that it could be used in our parallel study throughout three growing seasons [21]. With a good number of replicate plant individuals per species, we endeavored to understand how the polyphenol groups and activities show differences in their biological variation within each species. Still, the minimum requirement was to differentiate all species from each other by the help of the measured variables. To achieve this, we created a new mass spectrometric fingerprint mapping tool, which helps to visualize how the combination of qualitative and quantitative data of the selected polyphenol groups and related bioactivities can be used to effectively discriminate even closely related species of the same genus. Moreover, we normalized the quantitative polyphenol data with the larger data set that we have now acquired for <3500 species and <10,000 samples originating from five continents (Vanhakylä, Salminen et al., unpublished data). This way the visual x–y plots of our new fingerprint mapping tool were able to reveal the overall significance of the polyphenol groups and activities in each given species when compared against a wider presentation of the plant kingdom (see Results and Discussion for details).

## 2. Results and Discussion

Here, we tested how well the combined quantitation of eight most common subgroups of polyphenols (Figure 1) together with two polyphenol-driven activities—protein precipitation capacity and oxidative activity—can be used to reveal new types of species-specific patterns for a distinct set of 31 plant species. Replicates within the species were used to study the repeatability of the observed patterns. Below, we assess the results starting from the general patterns and moving towards the species-specific patterns.

### 2.1. General Patterns Observed from the Quantitative Data

**Figure 2:**
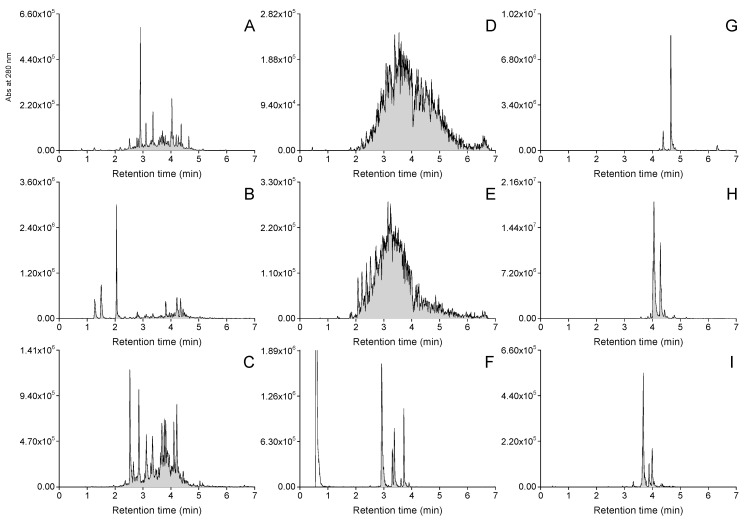
Examples of UHPLC-UV and group-specific UHPLC-MS/MS fingerprints recorded from the polyphenol extract of white birch (*Betula pubescens*). (**A**) UV traces at 280 nm, (**B**) galloyl derivative fingerprint, (**C**) hexahydroxydiphenoyl derivative fingerprint, (**D**) procyanidin polymer fingerprint, (**E**) prodelphinidin polymer fingerprint, (**F**) quinic acid derivative fingerprint, (**G**) kaempferol derivative fingerprint, (**H**) quercetin derivative fingerprint and (**I**) myricetin derivative fingerprint. The peak in panel F at 0.8 min represents free quinic acid found in plants, i.e., it is not a polyphenol.

The group-specific MS/MS tools were successfully able to reveal the intended polyphenol groups from the plant extracts containing them. Figure 2 shows examples of both the general UHPLC-UV and the UHPLC-MS/MS traces of the polyphenol groups detected in the foliage of *Betula pubescens*. The UHPLC-MS/MS chromatograms 2B–I show a peak or signal in each case when the group-specific MRM method has detected a specific functional unit in a compound separated using UHPLC and ionized using ESI-MS. Example chromatograms of all the studied species are presented in Figures S1–S31. Such group-specific chromatograms can reveal the diversity of individual compounds within the poly-phenol subgroups, showing that *B. pubescens* had higher diversity of HHDP derivatives (<30 peaks) than KA, QU or MY derivatives (>10 peaks). However, since the purpose of this study was not to focus on individual compounds per se, we quantified total contents of each of the detected subgroups in mg/g dry weight to highlight their proportional significance to the patterns observed in between species variation. The quantitation was achieved by integrating the detected 2D fingerprints as shown in Figure 2, and by converting the integrated total areas into concentrations with the help of calibration curves separately run for each compound group.

As expected, there was large between species variation in each of the phenolic categories detected by the group-specific MS/MS tools (Table S1). While all species did contain phenolics in general (3.4–229.0 mg/g DW), the quantitative threshold level was not exceeded for quinic acid derivatives in 11 species, for hydrolysable tannins in 17 species, for proanthocyanidins in seven species, and for protein precipitation capacity in four species. Interestingly, flavonol derivatives were found in all species, and this subgroup was dominated by quercetin and kaempferol derivatives, but myricetin derivatives were scarce in 17 species. Similar trends of variation within single polyphenol classes were observed for hydrolysable tannins (galloyl and HHDP groups) and proanthocyanidins (PC and PD groups) as well, suggesting that G/HHDP and PC/PD ratios could be good chemotaxonomic indicators together with the flavonol-based QU/KA/MY ratios.

### 2.2. Using the General Patterns to Create a 2D Fingerprint Mapping Tool

**Figure 3:**
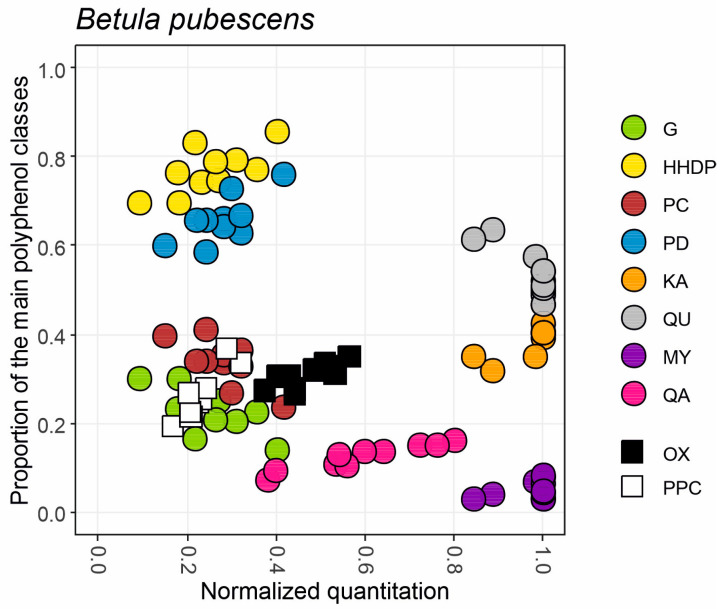
An example of a fingerprint map obtained for the main polyphenol groups and two bioactivities found in leaves of ten trees from a *B. pubescens* population. *X*-axis shows the normalized concentrations of hydrolysable tannins (G+HHDP), proanthocyanidins (PC+PD), flavonol derivatives (KA+QU+MY) and quinic acid derivatives (QA) together with oxidative activity (OX) and protein precipitation capacity (PPC) separately for each of the trees. *Y*-axis shows the proportions of the subgroups belonging to the main polyphenol groups (G/HHDP, PC/PD, KA/QU/MY) or proportions of QA, OX and PPC of the total phenolic levels recorded in the sample.

To take advantage of the observed patterns within the polyphenol classes and to use them more rapidly in species-to-species comparisons, we developed a 2D fingerprint mapping approach (Figure 3). This visual tool uses both quantitative (*x*-axis) and qualitative data (*y*-axis) to visually highlight the relative abundance of all measured polyphenol groups in a species. First, we normalized the quantitative data on the *x*-axis separately for each major polyphenol class (hydrolysable tannins, proanthocyanidins, flavonol derivatives and quinic acid derivatives) and activity (oxidative activity and protein precipitation capacity) by accounting the variation observed in our larger plant screening experiment including thousands of species from five continents (Vanhakylä, Salminen et al., unpublished data; see Materials and Methods for details). Second, we calculated the proportions of the polyphenol subgroups within these main classes to obtain the G/HHDP, PC/PD and QU/KA/MY ratios. The ratios of quinic acid derivatives (QA/TP) and the two activities (OX/TP and PPC/TP) were calculated against the total phenolic (TP) levels, since quantitative levels of QA, OX and PPC are in fact a part of TP.

For instance, Figure 3 shows how six out of ten *B. pubescens* trees had 1.0 levels of flavonol derivatives (grey, orange and purple dots); these trees belonged to the best 5% of flavonol-producing plants in the larger species set. The other four *B. pubescens* trees had slightly lower (0.84–0.95) normalized levels of flavonols. Flavonols of these 10 trees were >50% quercetin, ~40% kaempferol and <10% myricetin derivatives. Similarly, *B. pubescens* trees had 0.1–0.4 normalized levels of both hydrolysable tannins (yellow and green dots) and proanthocyanidins (blue and brown dots). HTs were dominated by HHDP derivatives (70–80%) over G derivatives (20–30%), and PAs by PDs (60–80%) over PCs (20–40%). These samples had proportionally similar levels of oxidative activity (black squares) and protein precipitation capacity (white squares), i.e., 20–40% proportion of total phenolics. However, the normalized score was a little higher for oxidative activity, suggesting that in a bigger picture *B. pubescens* leaves may produce compounds with better oxidative activity than protein precipitation capacity. This is in line with higher proportional levels of HHPD over G and PD over PC, since our previous studies have shown how both HHDP and PD groups are more important than G and PC for good oxidative activity [16,18,19,20]. Flavonols did not cause this pattern, since only myricetin derivatives have good oxidative activity and they produced <10% of the total flavonols [19]. Quinic acid derivatives showed quite a large quantitative variation (0.4–0.8; pink dots) and comprised a decent 10–20% share of total phenolics. All the other group-specific variables but QA derivatives were quite well grouped within the ten *B. pubescens* trees, showing that species-specific average values would have produced reliable quantitative and qualitative patterns for G+HHDP, G/HHDP, PC+PD, PC/PD, QU+KA+MY and QU/KA/MY, at least for this species. This way a single x–y graph was able to reveal the quantitative and qualitative nature of the main polyphenol classes and related activities in *B. pubescens* trees. In addition, the normalized quantitation allowed us to link the polyphenol levels of *B. pubescens* to those of thousands of other species in the plant kingdom and to draw conclusions on the polyphenol-based bioactivity in leaves of *B. pubescens*.

### 2.3. Main Patterns Revealed by the 2D Fingerprint Mapping

**Figure 4:**
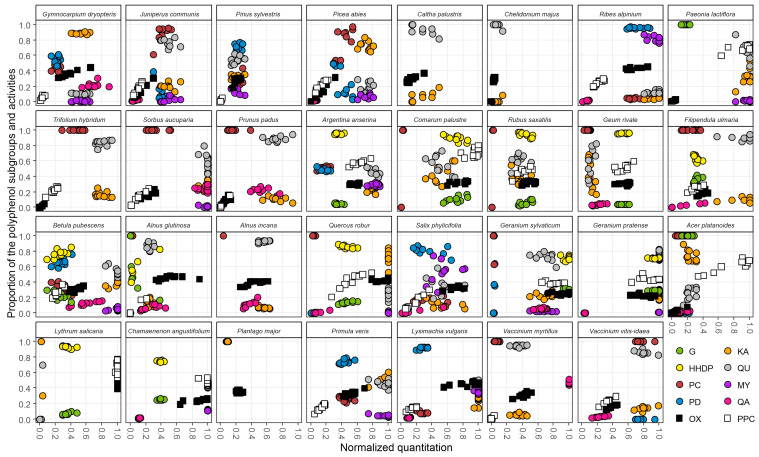
The variation for the 31 plant species in the fingerprint maps of their polyphenol subgroups and bioactivities (G, gallic acid derivatives; HHDP, hexahydroxydiphenoyl derivatives; PC, procyanidin units; PD, prodelphinidin units; KA, kaempferol derivatives; QU, quercetin derivatives; MY, myricetin derivatives; QA, quinic acid derivatives; OX, oxidative activity; PPC, protein precipitation capacity), as measured from the leaves, needles or flowers of ten individual plants. The *x*-axis shows the normalized concentrations of hydrolysable tannins (G+HHDP), proanthocyanidins (PC+PD), flavonol derivatives (KA+QU+MY), quinic acid derivatives, oxidative activity and protein precipitation capacity. The *y*-axis shows the proportions of the subgroups belonging to these main polyphenol groups (G/HHDP, PC/PD, KA/QU/MY) or the proportions of QA, OX and PPC of the total phenolic levels recorded in the sample.

Figure 4 shows species-specific polyphenol and bioactivity data of all the 31 species with 10 replicates in one graph. Species are organized as in Table S1 in phylogenetic order, i.e., ferns and gymnosperms (four species) are shown before the angiosperms (27 species). The phylogenetic tree of the studied species is represented in Figure S32 [22,23,24]. The clearest difference between these phylogenetic groups is the presence of hydrolysable tannins (G and HHDP) only in angiosperms (in 14 of the 27 species [25,26]). The evolutionarily older group of tannins, i.e., proanthocyanidins, was detected in the only fern and in all the three gymnosperms and in 20 of the angiosperms. Both of these two major tannin groups were simultaneously present in ten angiosperms and showed interesting variation both in their total content, but also in their qualitative PC/PD and G/HHDP ratios.

Quantitatively, nine of the 24 PA-containing species had low normalized levels (<0.2) of PAs, and even 15 species had lower than intermediate (<0.4) PA levels. Only two species had higher than intermediate levels of PAs (>0.6), i.e., *Ribes alpinum* and *Vaccinium vitis-idaea*. Qualitatively PAs were dominated by the PC units: in 13 species, the PC/PD ratio was higher than 95/5. Only in seven species, the PC/PD ratio was in the favor of the PDs (from 45/55 to 5/95).

For hydrolysable tannins, low levels (<0.2) were detected only in *Alnus glutinosa* and higher than intermediate levels (>0.6) in *Comarum palustre*, *Geranium sylvaticum* and *Geranium pratense*. As many as ten of the 14 HT-producing species were dominated by HHDP (>0.6) rather than galloyl derivatives, while two species (*Paeonia lactiflora* and *Acer platanoides*) contained only galloyl derivatives.

Quinic acid derivatives were the next biggest factor discriminating the species on the basis of their presence/absence data. They were present in 20 species: low levels (<0.2) were detected in six species and higher than intermediate levels (>0.6) in five species (*Gymnocarpium dryopteris*, *Sorbus aucuparia*, *B. pubescens*, *G. sylvaticum* and *Vaccinium myrtillus*).

Flavonol derivatives were the most common group of polyphenols as they were found in all the 31 species studied. They were typically richer in quercetin (12 species) or kaempferol derivatives (eight species) rather than myricetin derivatives (two species; *R. alpinum* and *Salix phylicifolia*). The remaining nine species contained rather equal amounts of two of the three flavonol groups without any group being the dominant one.

The 27 species showed both bioactivity types, oxidative activity and protein precipitation capacity. Only three species were dominated by normalized PPC over OX, while 14 were dominated by OX over PPC. This pattern together with the lack of PPC in four species (these species also lack HTs and PAs) and the presence of OX in all species suggested that OX is more uniformly distributed across many types of polyphenol groups while PPC is strictly linked with tannins only.

### 2.4. Specific Patterns Revealed by the 2D Fingerprint Mapping

The uneven distribution of the above main patterns between the species suggested that the 2D fingerprint mapping shown in Figure 4 can be used to find many patterns specific to plant species or to their evolutionary position in the plant phylogeny, or both. Next, we assess these patterns in more detail and link also the species polyphenol composition to their observed bioactivity levels. Where possible, we use our compound-level knowledge of species polyphenols to provide more insights into the basis of their bioactivities.

#### 2.4.1. Links of Oxidative Activity to the Observed Patterns of Polyphenol Groups

We have earlier revealed the main polyphenol groups important for plant oxidative activity: ellagitannins in general and especially the C-glycosidic ones [16,20,27] and PD-rich PAs together with myricetin-type flavonol glycosides [18,19,28]. All these polyphenol groups contain pyrogallol functions in their structures, making it the most critical and general phenolic moiety found in the most easily oxidized polyphenols. The other important phenolic feature for oxidative activity is the catechol group when it is found, e.g., in phenyl propanoids such as chlorogenic acid or phenyl ethanoids such as plantamajoside [28].

Indeed, looking at the Figure 4, it is evident that the majority of the intermediate (0.4–0.6) or high (>0.8) oxidative activity levels are linked to the presence of intermediate or high levels of HHDP derivatives. *Argentina anserina* and *C. palustre* share agrimoniin, a dimeric ellagitannin, as their main polyphenol [27,29,30], while *Geum rivale* has the dimeric ET gemin A [27] and *Rubus saxatilis* has the dimeric ET sanquiin H-6 and trimeric ET lambertianin C [27] as their main polyphenols. All these, just like the oligomeric ETs of *Chamaenerion angustifolium* (dimeric ET oenothein B and trimeric ET oenothein A, [27,31]), are glucopyranose-based ETs that are much less oxidatively active than ETs with acyclic glucose cores. Such acyclic ETs can be found in *Quercus robur* (monomeric vescalagin, castalagin and especially active castavaloninic acid [27,32,33]), but also in *Lythrum salicaria* (monomeric vescalagin and castalagin and dimeric salicarinins A, B, C and D [27,34,35,36]). In these two latter species, the oxidative activity levels were much higher than could be anticipated from their HHDP levels, suggesting that their specific HHDP derivatives contribute much more to the oxidative activity than HHDP derivatives of the other species. In contrast, both species of the genus *Geranium* had close to maximum levels of oxidative activity. That was simply due to the huge levels of monomeric glucopyranose-based ETs such as geraniin in their tissue, since sometimes a bit poorer compound-specific activity can be compensated by unusually high levels of the less active compounds in the plant tissue [37,38,39]. *P. lactiflora* and *A. platanoides* on the other hand were relatively poor in oxidative activity, since their gallotannins such as hexa-, hepta- and octagalloylglucoses have been shown to have a low oxidative activity in comparison to ellagitannins [20]. Interestingly, these two species did not contain any ellagitannins, but only gallotannins, which seems to be typical for gallotannin-producing species or plant organs. These two tannin types share the same immediate biosynthetic precursor pentagalloylglucose that functions as the branching point for gallotannin and ellagitannin biosynthesis [40]. A plant species or organ cannot thus produce high quantities of both of these tannin types at the same time.

Looking more closely at the tannin biosynthesis and its evolution, ferns and gymnosperms produced only PAs. *P. sylvestris* and *G. dryopteris* had higher PD content and thus also higher OX activity than did the more PC-rich *Juniperus communis* and *Picea abies*. *G. dryopteris* was the only one of these evolutionarily older plant lineages that reached intermediate OX levels, much because its QA derivatives that were represented by caffeoyl quinic acids with good oxidative activity [28].

The three angiosperms (*R. alpinum*, *S. phylicifolia* and *Lysimachia vulgaris*) that had higher than intermediate levels (>0.6) of oxidative activity without the ability to produce ETs either, accumulated even 80–95% PDs over PCs in their PAs. The good overall activity of pyrogallol over catechol functions was further highlighted by the relatively high levels of myricetin glycosides in all these three species. Moreover, the main polyphenol in *S. phylicifolia* is the flavanol dihydromyricetin [1,18,28,41,42] that has the same oxidatively active pyrogallol function that is seen in all myricetin-type flavonols. Obviously, its dihydrated structure (MW 320 g/mol) will not be detected by the myricetin-specific MS/MS detection tools (myricetin’s MW is 318 g/mol), but its presence can be easily witnessed by its UV spectrum typical to flavanols and the main *m/z* values of 319 ([M-H]^−^ ion) and 639 ([2M-H]^−^ion) from the full scan MS data that is simultaneously acquired with the group-specific MS/MS chromatograms. This is just to remind how simultaneous detection of UV and mass spectra can help in the polyphenol characterization [43], especially when the automated group-specific MS/MS detection tools miss some of the main compounds not belonging to the polyphenol groups to be detected.

In cases where HHPD, PD or MY derivatives were not able to explain the relatively high oxidative activity, or higher than suggested by the co-occurrence of these three polyphenol groups, the activity was linked to catechol-containing phenylpropanoids or phenylethanoids. For instance, *A. glutinosa* and *A. incana* are known to contain diarylheptanoids [5,44] with the active phenylethanoid group, and their major polyphenols oregonin in *A. glutinosa* (*m/z* 477) and rubranoside A in *A. incana* (*m/z* 493) can be spotted by their characteristic UV and mass spectra as well, although not detected by the group-specific MS/MS tools not designed for their detection.

In three out of the 31 species, the automated MS/MS tools detected only flavonol-type polyphenols, with myricetin derivatives being at very low levels. Still, these species are known to contain polyphenols that have structural functions, which we showed above, are required to possess oxidative activity. *P. major* is known for its main compound plantamajoside [45] that contains both caffeoyl and phenylethyl moieties. *C. palustris* contains fukinolic acid with a caffeoyl and a phenylethyl-like moieties [28]. *Chelidonium majus* is known for its alkaloid content [46,47], but contains oxidatively active compounds such as caffeoyl hexaric acid, caffeoyl threonic acid and caffeoyl malic acid [28].

#### 2.4.2. Links of Protein Precipitation to the Observed Patterns of Polyphenol Groups

If the oxidative activity of plant extracts depended much on their specific tannin composition, the protein precipitation capacity was non-surprisingly driven even more heavily by tannins. However, the patterns observed for these two types of activities did not vary in concert with the tannin content, since specific tannins may have high oxidative activity, but low protein precipitation capacity or vice versa [14,16,48]. For instance, *P. lactiflora* and *A. platanoides* with high levels of gallotannins achieved really good PPC scores, although their OX levels were really poor. This is in line with the good PPC of gallotannins that is due to especially their flexible galloyl groups and their relatively large size in comparison to monomeric ETs. In general, molecular size and flexibility are two of the most critical factors increasing the PPC of tannins [14,49,50].

By looking at the location of the PPC boxes together with the G+HHDP and PC+PD boxes in the x–y plots of Figure 4, one can easily make a few key notes. For instance, in ferns and gymnosperms, the PPC values are always a bit lower than the PC+PD values, suggesting that these species do not have optimal tannins for PPC. The same pattern is repeated for all of the angiosperms that produce PAs, but miss HTs. In contrast, only for 2/14 of the angiosperms producing HTs, the PPC boxes are located at a weaker normalized quantitative area than their normalized G+HHDP levels. These are the two *Geranium* species that produce monomeric geraniin-type DHHDP esters (ellagitannins) that are known for their relatively poor PPC [14]. In this respect, both *L. salicaria* and *C. angustifolium* represent the other end of the activity versus concentration spectrum as their normalized PPC hugely outperforms their normalized G+HHDP levels. This is presumably due to the salicarinin-type dimeric ellagitannins in *L. salicaria* [14,34,35,36] and the largest oligomeric ellagitannins of the plant kingdom present in *C. angustifolium* (oenothein-type ellagitannins ranging from dimers to undecamers [48,51,52]). In our in vitro PPC experiments with pure ETs, the high PPC of salicarinins was surprising, since they are composed of C-glycosidic monomers. Such acyclic monomeric ETs are rigid and have lost part of their flexibility due to the formation of HHDP and NHTP groups from two and three adjacent galloyl groups, respectively. However, when one such rigid monomer with a HHDP and NHTP group (vescalagin or castalagin) is attached to a corresponding monomer with one free and flexible galloyl group and two HHDP groups (stachyurin or casuarinin) via an *m*-DOG type linkage, the formed dimer is again flexible. This is due to the intramolecular *m*-DOG linkage that connects the flexible galloyl group of either stachyurin or casuarinin to the HHDP group of vescalagin or castalagin to form salicarinins A–D. This way these oligomers are both large and flexible and thus show good PPC. Examples of all these tannin structures have been earlier published in [14,15].

It is exciting that the above types of peculiar species-specific patterns with relatively low HHDP levels and relatively high PPC levels could be seen directly from the fingerprint maps and that they co-occurred in species with those highly PPC-active ellagitannins. These types of visual plots could thus form a good tool for rapid spotting of such species in the plant kingdom that seem to produce something exceptional in their reservoir of the OX or PPC-active polyphenols, i.e., higher proportion of bioactivities than expected on the basis of their measured group-specific polyphenol levels. At the same time, it can be noted that these patterns work also in the other direction, as shown for both *G. sylvaticum* and *G. pratense*. These species had a slightly lower normalized PPC than G+HHDP levels, suggesting that the tannins of these species have non-optimal structures for PPC; this was verified by the presence of DHHDP-type monomeric esters such as geraniin in these species [37,38].

If species contains both PPC and OX, their normalized levels can be used to find if the species is clearly better in either of these activities, when normalized over the large species set. While *P. lactiflora* and *A. platanoides* seemed to be effective in PPC, but relatively poor in OX, the reverse seemed to be true for at least *R. alpinum*, *S. phylicifolia*, *Primula veris* and *L. vulgaris*. All of these latter species produce PD-rich PAs, with as high as 70–95% PD-share, but none produce HTs. While high PD rather than PC share can be linked to better PPC [53], the same is true also for the oxidative activity of PAs [20]. Our visual fingerprint maps clearly suggest that high PD share has bigger impact on the OX than on the PPC of plants.

#### 2.4.3. Other Types of Patterns Emerging from the Fingerprint Maps

**Figure 5:**
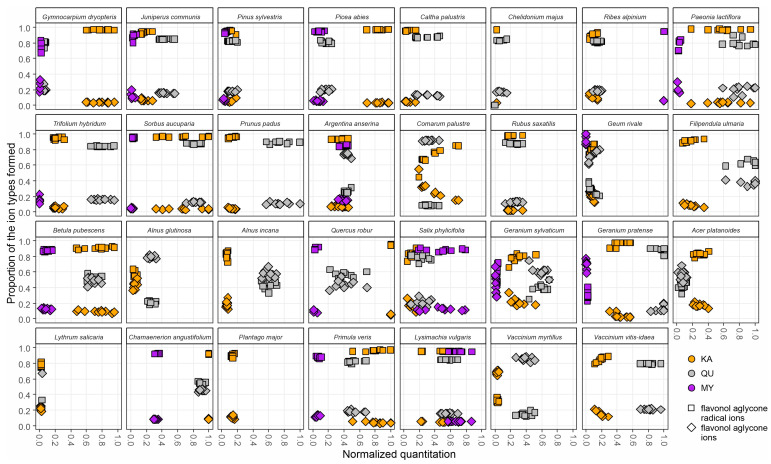
The variation for the 31 plant species in the detailed fingerprint maps of the three flavonol subgroups (KA, kaempferol derivatives; QU, quercetin derivatives; MY, myricetin derivatives) including the proportions of the aglycone radical ions (squares) and aglycone ions (diamonds) formed during the fragmentation of the original flavonol derivatives in the MS ion source before the specific MRM detection of the flavonol subgroups. The *x*-axis shows separately the normalized concentrations of KA, QU and MY derivatives in the ten replicate plants. The *y*-axis shows the proportions of the aglycone radical ions to the aglycone ions detected from the KA, QU and MY derivatives found in the corresponding plant individuals.

Some of the above G+HHDP and PC+PD patterns that contributed to the oxidative activity, but also patterns emerging from quercetin and kaempferol derivatives could be used either as species-specific chemotaxonomic markers or to witness plant and polyphenol co-evolution. To make these patterns even more clearly species-specific, we had an additional look at the ratios of flavonol aglycone ions (*m/z* 285, *m/z* 301, and *m/z* 317) to the flavonol aglycone ion radicals (*m/z* 284, *m/z* 300, and *m/z* 316) formed in the fragmentation of the flavonol glycosides in the ion source from kaempferol, quercetin and myricetin derivatives, respectively. In the method development paper, Engström et al. [12] showed how the ratio of these ions formed depends on the glycosylation pattern of the glycosides, e.g., pure 3-*O*-glycosides producing more of the aglycone ion radical and other glycosides producing more of the aglycone ion. However, this ratio is also affected by other decoration patterns of the flavonols such as the methylation of the OH-group in the B-ring of 3-*O*-glycosides; such methylation affects the ion ratio that is no longer in clear favor of the aglycone ion radical as it was in non-methylated 3-*O*-glycosides (Salminen, unpublished data). Nevertheless, the differences in the detected ratios of these ions between plant species show that their core flavonol aglycones are differently substituted. Figure 5 shows the separate normalized quantitation for each of the three flavonol subgroups at the *x*-axis, and the proportions of these two ion types formed at the *y*-axis. It can be seen that in some species the flavonol aglycone ion radicals are clearly favored suggesting the presence of 3-*O*-glycosides, while in others, flavonol aglycone ions are favored, suggesting the presence of 7-*O*-glycosides or 4′-*O*-glycosides, or these two ions types in nearly the same proportion. For instance, in *A. glutinosa* and *A. incana,* these two ion types were either grouped (KA in *A. glutinosa* and QU in *A. incana*) or separated (QA in *A. glutinosa* and KA in *A. incana*), suggesting different types of KA and QU derivatives in these *Alnus* species (Figure 5).

The Rosaceae plant family was most widely represented in this study. Five out of seven Rosaceae species contained HTs and were dominated by HHDP over galloyl derivatives. Of these, *Filipendula ulmaria* differed qualitatively from the four others by having 40/60 G/HHDP ratio while others had close to 10/90 ratio. *C. palustre* and *R. saxatilis* had somewhat similar polyphenol and activity maps (Figure 4), but their detailed flavonol maps (Figure 5) were very different. For instance, *R. saxatilis* contained kaempferol and quercetin derivatives that produced mainly flavonol aglycone ion radicals, while for *C. palustre,* this was true for kaempferol derivatives, but its quercetin derivatives produced mostly flavonol aglycone ions instead. Of the PA-producing Rosaceae species, *S. aucuparia* was well distinguished from *Prunus padus* by the flavonol and quinic acid derivatives (Figure 4) and by the detailed flavonol patterns (Figure 5). However, *P. padus* (Rosaceae) was very similar to *Trifolium hybridum* (Fabaceae) even from its flavonol patterns (Figure 5) and the only true difference was found in the quinic acid derivatives that were absent in *T. hybridum* but clearly present at 0.4–0.8 levels in *P. padus*. All these findings suggest that all the measured variables in Figure 4 and Figure 5 were important to create a difference between the species. Where such a difference could not be unequivocally obtained (only *C. palustris* versus *C. majus*), UV and full scan MS chromatograms enabled species separation by more laborious manual inspection.

There were other biosynthetically important patterns in addition to the lack of coexistence of gallotannins with ellagitannins. Both of these hydrolysable tannin types need gallic acid as their primary building block and it is produced from shikimic acid, the intermediate compound of the shikimate pathway [54]. At the same time, this biosynthetic flux of energy is directed away from the phenylpropanoid pathway, since it is located directly after the shikimate pathway. Since both flavonols and PAs need the phenylpropanoid pathway, their biosynthesis is affected by the production of high levels of gallic acid and HTs. For these reasons, none of the 31 species produced simultaneously higher than 0.4 normalized levels of both HTs and PAs. The normalized levels of flavonol production per se was not that heavily affected by HTs or PAs, since flavonol levels in general were approximately seven times lower than HTs and PAs. However, as shown for the flavonol types, myricetin derivatives were their most scarce group, but they co-existed especially with the high PD-levels (*R. alpinum*, *S. phylicifolia* and *L. vulgaris*). On one hand, one could have expected a negative correlation between these two compound groups as they have the same trihydroxy substitution in their B-ring and compete for the same biosynthetic precursors within the flavonoid pathway. On the other hand, it has been shown that the overexpressing of the transcription factors regulating PD synthesis in plants can lead to enhanced flavonoid B-ring hydroxylation and thus co-occurrence of high levels of both PD-rich PAs and myricetin derivatives [55]. Such could have been the case with *R. alpinum*, *S. phylicifolia* and *L. vulgaris*.

#### 2.4.4. Repeatability of the Patterns to Obtain Species-Specific Fingerprint Maps

All the patterns shown in Figure 4 and Figure 5 and discussed above are meaningless, if they are not reproducible. For this reason, we collected ten replicates for each species to record the within species variation in all the measured variables that are well highlighted in Figure 4 and Figure 5. Some of the variables had higher quantitative variation with very little qualitative variation. For instance, *P. padus* flavonols varied between 0.4–0.95 normalized levels, but the KA/QU/MY ratios always had 85–95% quercetin derivatives. The total opposite was observed in *G. rivale*, as the flavonols were at 0.1–0.2 normalized levels, but the KA/QU/MY varied from 30% to 80% quercetin derivatives. For some species like *A. anserina*, all replicates were really tightly packed for all measured variables, even the flavonols. Then again, *S. phylicifolia* was the only example where practically none of the measured variables formed clear clusters with the replicates. For the species itself, this could be a positive indication of species-specific plasticity in polyphenol production depending on needs. In general, Figure 4 and Figure 5 showed that the x–y scale fingerprint maps formed clear species-specific patterns for 30 out of the 31 of the species, excluding only *S. phylicifolia*.

## 3. Materials and Methods

### 3.1. Plant Sampling and Extraction

The plant samples were collected from Turku and nearby areas in South–West Finland during the summer 2016. Selected plant species and collection information are listed in Table S2. Each specimen was sampled from a mature plant after the most significant growing phase because the young plant’s polyphenol content can differ significantly from a fully-grown plant [56,57]. Only new-growth needles of *J. communis* and *P. abies* were sampled at relatively young stage in June. Ten specimens were sampled from the same population and each sample consisted of up to ten leaves from different parts of the plant to cover the variation within a plant specimen. Only *P. veris*, *C. angustifolium* and *T. hybridum* were studied for their flower chemistry. The samples were stored in aluminum foil in ice during the collection to slow down the enzymatic activity.

Samples were frozen overnight at −21 °C. All samples were lyophilized at least for 24 h, but typically for 48 h. The dry material was ground into a fine powder with a ball mill. A 20 mg quantity of plant powder was first mixed with 1400 µL of acetone/water (8/2, *v*/*v*) solvent in 2 mL Eppendorf tube and left to macerate overnight at 4 °C, and then extracted on a planary shaker for 3 h. After centrifugation (10 min, 14 000 rpm), the supernatant was collected into a new 2 mL Eppendorf tube and acetone was evaporated with an Eppendorf concentrator at room temperature. The extraction step was repeated once, the extracts were combined and the remaining acetone was evaporated with the concentrator. The aqueous phase was frozen and freeze-dried. The freeze-dried extract was dissolved in 1 mL of ultra-pure water and filtered with a 0.2 μm PTFE syringe filter before further analyses.

### 3.2. Bioactivity Measurements

#### 3.2.1. Oxidative Activity Measurement

Total phenolic content and oxidatively active phenolics were measured with the modified Folin–Ciocalteu assay as described in detail by Salminen and Karonen [48] using Multiscan Ascent microplate reader (Labsystems and Thermo Electron Corporation). In short, the method measures the phenolic content of both the initial and oxidized plant extracts as gallic acid equivalents, which provides both the total phenolic and oxidative activity levels of the original plant sample in mg/g dry weight.

The oxidation step was conducted in 96-well plates as follows. An aliquot of 20 µL of the previously prepared plant extract was pipetted on a plate in triplicate. Oxidation was initiated by adding 180 µL of sodium carbonate buffer (pH 10) and samples were incubated at 25 °C for 60 min, shaking for 10 s every min. After exactly 60 min, the oxidation was stopped by adding 100 µL of 0.6% formic acid into the wells. To obtain the total phenolic levels of the initial extract without oxidation, 20 µL of the initial extract was diluted with 280 µL of the buffer mixture containing 180 µL of sodium carbonate (pH 10) and 100 µL of 0.6% aqueous formic acid.

The total phenolic levels of both the oxidized and non-oxidized extracts were measured with the Folin–Ciocalteu assay. A gallic acid standard curve (prepared in water: 10 µg/mL, 25 µg/mL, 100 µg/mL, R^2^ = 0.99) was analyzed similarly to enable the TP determination. First, 50 µL of samples were pipetted on wells in triplicate, and 50 µL of Folin–Ciocalteu reagent was added. After a minute of shaking, 100 µL of 20% sodium carbonate (*m*/*v*) was added, and plate was shaken again. Immediately after shaking, the absorbance was measured at 1 min intervals at 742 nm for 30 min, and the maximal absorbance was used to calculate the total phenolic levels.

#### 3.2.2. Protein Precipitation Capacity Measurement

Protein precipitation capacity of plant samples was measured with the radial diffusion assay (RDA) [58], which is based on the ability of polyphenols and especially tannins to precipitate bovine serum albumin (BSA) protein on an agar gel to form detectable precipitation rings. A mixture of pentagalloylglucose/oenothein B (PGG/OeB) (1/1, *v*/*v*) was used as a standard to determine the concentration of polyphenols with the PPC activity.

First, the agar-BSA dishes were prepared. RDA buffer was prepared by adding 10.6 mg of ascorbic acid and 2.85 mL of glacier acetic acid into 1 L of ultra-pure water. The pH was set to 5 by adding 2 M NaOH. The agarose gel was prepared by adding 10.0 g of agarose into 1000 mL of heated RDA buffer (90 °C). After cooling to 45 °C, 1.0 g of BSA was added. Then, 10 mL of prepared solution was pipetted into Petri dishes and was allowed to set for 60 min. The dishes were stored in a cold room upside down before the next steps.

The plant extracts were 2× concentrated before RDA analyses by freeze-drying 200 µL of the extract that was then dissolved in 100 µL of ultra-pure water. A 24 µL volume of this extract was pipetted on three wells punched into the Petri dish. Each sample was pipetted in triplicate. PGG/OeB standards (1, 2, 3, 4 and 5 mg/mL in 30% aqueous EtOH) were similarly pipetted into the Petri dishes in triplicate. The solutions were let to diffuse into the gel for an hour; dishes were sealed with parafilm and incubated upside down in an oven (30 °C) for three days. The precipitation ring areas were measured using ImageJ image processing software [59] and transformed into concentration (mg/g dry weight) using the PGG/OeB standard curve (R^2^ value ranging from 0.83 to 0.99).

### 3.3. UHPLC-DAD-MS Analyses

Each of the ten replicate plant extracts were analyzed once with an ultrahigh-performance liquid chromatography system coupled with a diode array detector and triple quadrupole mass spectrometer (UHPLC-DAD-QqQ-MS). The UHPLC system consisted of a sample manager, a binary solvent manager, an Acquity UPLC^®^ BEH Phenyl column (100 mm × 2.1 mm i.d., 1.7 µm; Waters Corporation, Wexford, Ireland), a photodiode array detector and Xevo TQ triple quadrupole mass spectrometer (Waters Corporation, Milford, MA, USA). Analysis conditions were identical to the conditions described by Engström et al. [11,12]. The flow rate was 0.5 mL/min. Two eluents were used in the gradient elution, i.e., acetonitrile (A) and 0.1% aqueous formic acid (B), as follows: 0–0.5 min, 0.1% A in B; 0.5–5.0 min, 0.1–30% A in B (linear gradient); 5.0–6.0 min, 30–35% A in B (linear gradient); and 6.0–9.5 min, column wash and stabilization. UV and MS data were recorded from 0 to 7 min. MS analyses were conducted on a negative-ion mode. The capillary voltage was set at 2.4 kV, desolvation temperature 650 °C, source temperature 150 °C, desolvation and cone gas was N_2_ with flow rates 1000 L/h and 100 L/h and collision gas was argon.

Polyphenol subgroups were quantified using the group-specific MRM methods for galloyl and HHDP derivatives, quinic acid derivatives, kaempferol, quercetin and myricetin derivatives, procyanidins and prodelphinidins [11,12]. All the MRM transitions and methodological details are shown in the original publications [11,12]. Shortly, the group-specific methods first fragment the UHPLC-separated polyphenols in the ion source using cone voltages separately optimized for each polyphenol group. Then, the specific precursor ions representing the selected functional units of polyphenols are selected in the first quadrupole for fragmentation in the collision cell. The precursor-specific product ions are first accumulated by optimized collisions energies and then selected by the last quadrupole for detection and creation of the group-specific fingerprints (Figure 2). All of the fingerprints were smoothed and integrated with the TargetLynx software (Waters Corporation). External standards were used to transform the integrated group-specific fingerprints result into mg/g dry weight in each sample, and a catechin solution (5 µg/mL) was injected into the system before and after every ten samples to correct for any changes in the MS performance. The method and its use are described in detail in Engström et al. [11,12] and in Malisch et al. [60].

### 3.4. Mass Spectrometric Fingerprint Mapping

To map the qualitative and quantitative results of the eight polyphenol groups and the two bioactivities with different variability scales on a same comprehensive chart, the data was first normalized between zero and 1.0 using a selected maximal value for each polyphenol and activity group. Maximal values were determined accordingly to Natural Chemistry Research Group’s plant library (<10,000 samples, <3500 species, Vanhakylä, Salminen et al. unpublished). This allows us to compare any data set to the occurrence of the selected polyphenol groups overall in the plant kingdom. To highlight and balance the contrast at lower concentrations, the maximal level was set to cover 95% of the samples, equalizing 5% of the highest values to the maximal level 1.0. The highest 5% of the data was scattered on a large concentration scale, and if the absolutely maximal concentrations were given the value of 1.0, most of the samples would have been grouped at the low end of the concentration axis.

The maximal values (mg/g dry weight) for normalization were set as following; OX: 40.0; PPC: 70.0; HT: 85.0; PA: 85.0; FL: 10.0; QA: 13.0; GA: 25.0; HHDP: 75.0; PC: 75.0; PD 45.0; KA: 6.0; QU: 8.5 and MY: 6.0. The initial concentrations at the normalized points and detected maximal values of each polyphenol and activity group are listed in Table S3. The initial concentrations of each subgroup of flavonol derivatives at the normalized scale are listed in Table S4.

The quantification limit was set to 0.1 mg/g for flavonol glycosides, hydrolyzable tannins and quinic acid derivatives and to 1.0 mg/g for proanthocyanidins. These corresponded approx. to 5 × 10^4^ levels of detection per their MRM trace. The higher quantification limit of PAs was due to their characteristic way to form oligomer and polymer humps in chromatograms, making the integrated areas broader and thus the detection limits higher in mg/g than with the other compound types producing defined sharp peaks. By these relatively high quantification limits, we aimed to avoid reporting false-positive values for any of the species. Small levels (a few mg/g) of false positives are possible with certain MRM methods especially when the polyphenols causing false positives are in a high proportion. For instance, already the method development paper [12] showed that quercetin derivatives produce a 1% false-positive detection in the HHDP-specific MRM method (the functional units quercetin and HHDP have the same *m/z* value of 301 when fragmented from the original compound). For this reason, 1% of the integrated quercetin-specific fingerprint area (Figure S19H) must be omitted from the integrated HHDP-specific fingerprint area (Figure S19C). This way false HHDP positives are not reported, and the HHDP levels are more correct even for the truly HHDP-containing species.

## 4. Conclusions

In this study, we created a new visual fingerprint mapping tool and showed how the qualitative and quantitative data of eight common polyphenol groups alone were able to discriminate 31 plant species from each other. This could be seen from the fingerprint maps including either all the measured polyphenol groups and activities (Figure 4) or only the detected flavonol derivatives (Figure 5). This finding highlights a few key issues regarding the use of plant polyphenols as chemotaxonomic marker compounds as their wide presence in the plant kingdom can be either a benefit or a disadvantage, depending on how they are analyzed. For instance, we found flavonols in all species and tannins in all but three species, showing that these two rough polyphenol groups per se are not necessarily good candidates as chemotaxonomic indicators. However, when the biosynthetically linked flavonols were divided into three subgroups (KA, QU, MY) depending on the hydroxylation pattern of their B-ring, the situation changed dramatically. Similarly, when tannins were divided into two biosynthetically distinct groups (HTs and PAs) and these further into two biosynthetically linked subclasses, the G/HHDP and PC/PD ratios together with the normalized PA and HT content quite efficiently separated the tannin-containing species from each other. Finally, the normalized levels of the two types of bioactivities—protein precipitation and oxidative activity—showed quite a variable distribution in the fingerprint maps being even able to reveal species with unusually efficient poly-phenols. It will be interesting to see how well this tool is able to separate tens of species in a plant genus or hundreds of species in a plant family and if new polyphenol types with promising activities can be found in species not yet analyzed for their detailed polyphenol chemistry.

## Data Availability

The data presented in this study is available on request from the corresponding author.

## Supplementary Materials

The following supporting information can be downloaded at: https://www.mdpi.com/article/10.3390/molecules28176388/s1, Figure S1–S31. Examples of UHPLC-UV and group-specific UHPLC-MS/MS fingerprints recorded from the polyphenol extract of each species. (A) UV traces at 280 nm, (B) galloyl derivative fingerprint, (C) hexahydroxydiphenoyl derivative fingerprint, (D) procyanidin polymer fingerprint, (E) prodelphinidin polymer fingerprint, (F) quinic acid derivative fingerprint (the peak in at 0.8 min is free quinic acid found in plants, i.e., it is not a polyphenol), (G) kaempferol derivative fingerprint, (H) quercetin derivative fingerprint and (I) myricetin derivative fingerprint. The y-axes are scaled to the most intensive peak of each fingerprint. Figure S32: Phylogenetic tree of 31 plant species in relative time scale. Table S1. The initial concentrations (mg/g, dry weight) of the polyphenol classes and bioactivities of the studied plant populations. The concentration values are population averages ± standard error of the mean. ND: not detected, NS: not significant. Table S2: Collected plant species, their growth forms, collected organs, collection dates and coordinates. The species are listed in a phylogenetic order presented in Figure S32. Table S3: The initial concentration values (mg/g) of each polyphenol group and bioactivity at normalized scale (0.0–1.0), detection limit (0.0) and detected maximal values (max). Table S4: The initial concentration values (mg/g) of each flavonol group and glycosylation pattern at normalized scale. References [22,23,24] are cited in the Supplementary Materials.

## References

[1] Manninen M., Karonen M., Salminen J.-P. (2022). Chemotaxonomic Markers for the Leaf Buds of Common Finnish Trees and Shrubs: A Rapid UHPLC MS Fingerprinting Tool for Species Identification. Molecules.

[2] Lahtinen M., Lempa K., Salminen J.-P., Pihlaja K. (2006). HPLC Analysis of Leaf Surface Flavonoids for the Preliminary Classification of Birch Species. Phytochem. Anal..

[3] Julkunen-Tiitto R. (1989). Phenolic Constituents of *Salix*: A Chemotaxonomic Survey of Further Finnish Species. Phytochemistry.

[4] Giffen J.E., Lesiak A.D., Dane A.J., Cody R.B., Musah R.A. (2016). Rapid Species-Level Identification of Salvias by Chemometric Processing of Ambient Ionisation Mass Spectrometry-Derived Chemical Profiles. Phytochem. Anal..

[5] Vidakovic V., Novakovic M., Popovic Z., Jankovic M., Matic R., Teševic V., Bojovic S. (2017). Significance of Diarylheptanoids for Chemotaxonomical Distinguishing between *Alnus glutinosa* and *Alnus incana*. Holzforschung.

[6] Van Der Hooft J.J.J., Akermi M., Ünlü F.Y., Mihaleva V., Roldan V.G., Bino R.J., De Vos R.C.H., Vervoort J. (2012). Structural Annotation and Elucidation of Conjugated Phenolic Compounds in Black, Green, and White Tea Extracts. J. Agric. Food Chem..

[7] Ridder L., Van Der Hooft J.J.J., Verhoeven S., De Vos R.C.H., Bino R.J., Vervoort J. (2013). Automatic Chemical Structure Annotation of an LC-MS^n^ Based Metabolic Profile from Green Tea. Anal. Chem..

[8] Wolfender J.L., Nuzillard J.M., Van Der Hooft J.J.J., Renault J.H., Bertrand S. (2019). Accelerating Metabolite Identification in Natural Product Research: Toward an Ideal Combination of Liquid Chromatography-High-Resolution Tandem Mass Spectrometry and NMR Profiling, in silico Databases, and Chemometrics. Anal. Chem..

[9] Rutz A., Sorokina M., Galgonek J., Mietchen D., Willighagen E., Gaudry A., Graham J.G., Stephan R., Page R., Vondrášek J. (2022). The LOTUS Initiative for Open Knowledge Management in Natural Products Research. elife.

[10] Allard P.M., Gaudry A., Quirós-Guerrero L.M., Rutz A., Dounoue-Kubo M., Walker T.W.N., Defossez E., Long C., Grondin A., David B. (2022). Open and Reusable Annotated Mass Spectrometry Dataset of a Chemodiverse Collection of 1600 Plant Extracts. Gigascience.

[11] Engström M.T., Pälijärvi M., Fryganas C., Grabber J.H., Mueller-Harvey I., Salminen J.-P. (2014). Rapid Qualitative and Quantitative Analyses of Proanthocyanidin Oligomers and Polymers by UPLC-MS/MS. J. Agric. Food Chem..

[12] Engström M.T., Pälijärvi M., Salminen J.-P. (2015). Rapid Fingerprint Analysis of Plant Extracts for Ellagitannins, Gallic Acid, and Quinic Acid Derivatives and Quercetin-, Kaempferol- and Myricetin-Based Flavonol Glycosides by UPLC-QqQ-MS/MS. J. Agric. Food Chem..

[13] Salminen J.-P. (2018). Two-Dimensional Tannin Fingerprints by Liquid Chromatography Tandem Mass Spectrometry Offer a New Dimension to Plant Tannin Analyses and Help to Visualize the Tannin Diversity in Plants. J. Agric. Food Chem..

[14] Engström M.T., Arvola J., Nenonen S., Virtanen V.T.J., Leppä M.M., Tähtinen P., Salminen J.-P. (2019). Structural Features of Hydrolyzable Tannins Determine Their Ability to Form Insoluble Complexes with Bovine Serum Albumin. J. Agric. Food Chem..

[15] Engström M.T., Virtanen V., Salminen J.-P. (2022). Influence of the Hydrolyzable Tannin Structure on the Characteristics of Insoluble Hydrolyzable Tannin-Protein Complexes. J. Agric. Food Chem..

[16] Moilanen J., Salminen J.-P. (2008). Ecologically Neglected Tannins and Their Biologically Relevant Activity: Chemical Structures of Plant Ellagitannins Reveal Their in vitro Oxidative Activity at High pH. Chemoecology.

[17] Engström M.T., Sun X., Suber M.P., Li M., Salminen J.-P., Hagerman A.E. (2016). The Oxidative Activity of Ellagitannins Dictates Their Tendency to Form Highly Stabilized Complexes with Bovine Serum Albumin at Increased pH. J. Agric. Food Chem..

[18] Vihakas M., Pälijärvi M., Karonen M., Roininen H., Salminen J.-P. (2014). Rapid Estimation of the Oxidative Activities of Individual Phenolics in Crude Plant Extracts. Phytochemistry.

[19] Kim J., Pälijärvi M., Karonen M., Salminen J.-P. (2018). Oxidatively Active Plant Phenolics Detected by UHPLC-DAD-MS after Enzymatic and Alkaline Oxidation. J. Chem. Ecol..

[20] Barbehenn R.V., Jones C.P., Hagerman A.E., Karonen M., Salminen J.-P. (2006). Ellagitannins Have Greater Oxidative Activities than Condensed Tannins and Galloyl Glucoses at High pH: Potential Impact on Caterpillars. J. Chem. Ecol..

[21] Vanhakylä S., Salminen J.-P. (2023). Seasonal Variation in Plant Polyphenols and Related Bioactivities Across Three Years in Ten Tree Species as Visualized by Mass Spectrometric Fingerprint Mapping. Molecules.

[22] Webb C.O., Donoghue M.J. (2005). Phylomatic: Tree Assembly for Applied Phylogenetics. Mol. Ecol. Notes.

[23] Zanne A.E., Tank D.C., Cornwell W.K., Eastman J.M., Smith S.A., FitzJohn R.G., McGlinn D.J., O’Meara B.C., Moles A.T., Reich P.B. (2014). Three Keys to the Radiation of Angiosperms into Freezing Environments. Nature.

[24] Letunic I., Bork P. (2021). Interactive Tree of Life (ITOL) v5: An Online Tool for Phylogenetic Tree Display and Annotation. Nucleic Acids Res..

[25] Bate-Smith E.C. (1977). Astringent tannins of Acer species*. Phytochemistry.

[26] Haslam E. (1988). Plant Polyphenols (Syn. Vegetable Tannins) and Chemical Defense-A Reappraisal. J. Chem. Ecol..

[27] Moilanen J., Koskinen P., Salminen J.-P. (2015). Distribution and Content of Ellagitannins in Finnish Plant Species. Phytochemistry.

[28] Kim J., Pälijärvi M., Karonen M., Salminen J.-P. (2020). Distribution of Enzymatic and Alkaline Oxidative Activities of Phenolic Compounds in Plants. Phytochemistry.

[29] Olennikov D.N., Kashchenko N.I., Chirikova N.K., Kuzmina S.S. (2015). Phenolic Profile of *Potentilla anserina* L. (Rosaceae) Herb of Siberian Origin and Development of a Rapid Method for Simultaneous Determination of Major Phenolics in *P. anserina* Pharmaceutical Products by Microcolumn RP-HPLC-UV. Molecules.

[30] Kashchenko N.I., Chirikova N.K., Olennikov D.N. (2017). Agrimoniin, an Active Ellagitannin from *Comarum palustre* Herb with Anti-α-Glucosidase and Antidiabetic Potential in Streptozotocin-Induced Diabetic Rats. Molecules.

[31] Granica S., Piwowarski J.P., Czerwińska M.E., Kiss A.K. (2014). Phytochemistry, Pharmacology and Traditional Uses of Different *Epilobium* Species (Onagraceae): A Review. J. Ethnopharmacol..

[32] Salminen J.-P., Roslin T., Karonen M., Sinkkonen J., Pihlaja K., Pulkkinen P. (2004). Seasonal Variation in the Content of Hydrolyzable Tannins, Flavonoid Glycosides, and Proanthocyanidins in Oak Leaves. J. Chem. Ecol..

[33] Mayer W., Gabler W., Riester A., Korger H. (1967). Über Die Gerbstoffe Aus Dem Holz Der Edelkastanie Und Der Eiche, II. Die Isolierung von Castalagin, Vescalagin, Castalin Und Vescalin. Justus Liebigs Ann. Chem..

[34] Piwowarski J.P., Kiss A.K. (2013). C-Glucosidic Ellagitannins from Lythri Herba (*European Pharmacopoeia*): Chromatographic Profile and Structure Determination. Phytochem. Anal..

[35] Granica S., Piwowarski J.P., Kiss A.K. (2014). Determination of C-Glucosidic Ellagitannins in *Lythri salicariaeherba* by Ultra-High Performance Liquid Chromatography Coupled with Charged Aerosol Detector: Method Development and Validation. Phytochem. Anal..

[36] Piwowarski J.P., Granica S., Kiss A.K. (2015). *Lythrum salicaria* L.—Underestimated Medicinal Plant from European Traditional Medicine. A Review. J. Ethnopharmacol..

[37] Tuominen A. (2013). Defensive Strategies in *Geranium sylvaticum*, Part 2: Roles of Water-Soluble Tannins, Flavonoids and Phenolic Acids against Natural Enemies. Phytochemistry.

[38] Tuominen A., Salminen J.-P. (2017). Hydrolyzable Tannins, Flavonol Glycosides, and Phenolic Acids Show Seasonal and Ontogenic Variation in *Geranium sylvaticum*. J. Agric. Food Chem..

[39] Tuominen A., Toivonen E., Mutikainen P., Salminen J.-P. (2013). Defensive Strategies in *Geranium sylvaticum*. Part 1: Organ-Specific Distribution of Water-Soluble Tannins, Flavonoids and Phenolic Acids. Phytochemistry.

[40] Gross G.G., Romeo J.T. (1999). Biosynthesis, Biodegradation, and Cellular Localization of Hydrolyzable Tannins. Phytochemicals in Human Health Protection, Nutrition, and Plant Defense. Recent Advances in Phytochemistry.

[41] Tegelberg R., Veteli T., Aphalo P.J., Julkunen-Tiitto R. (2003). Clonal Differences in Growth and Phenolics of Willows Exposed to Elevated Ultraviolet-B Radiation. Basic Appl. Ecol..

[42] Rank N.E., Köpf A., Julkunen-Tiitto R., Tahvanainen J. (1998). Host Preference and Larval Performance of the Salicylate-Using Leaf Beetle *Phratora vitellinae*. Ecology.

[43] Moilanen J., Sinkkonen J., Salminen J.-P. (2013). Characterization of Bioactive Plant Ellagitannins by Chromatographic, Spectroscopic and Mass Spectrometric Methods. Chemoecology.

[44] Ren X., He T., Chang Y., Zhao Y., Chen X., Bai S., Wang L., Shen M., She G. (2017). The Genus *Alnus*, a Comprehensive Outline of Its Chemical Constituents and Biological Activities. Molecules.

[45] Ravn H., Brimer L. (1988). Structure and Antibacterial Activity of Plantamajoside, a Caffeic Acid Sugar Ester from *Plantago major* subsp Major. Phytochemistry.

[46] Sárközi Á., Janicsák G., Kursinszki L., Kéry Á. (2006). Alkaloid Composition of *Chelidonium majus* L. Studied by Different Chromatographic Techniques. Chromatographia.

[47] Gañán N.A., Dias A.M.A., Bombaldi F., Zygadlo J.A., Brignole E.A., De Sousa H.C., Braga M.E.M. (2016). Alkaloids from *Chelidonium majus* L.: Fractionated Supercritical CO_2_ Extraction with Co-Solvents. Sep. Purif. Technol..

[48] Salminen J.-P., Karonen M. (2011). Chemical Ecology of Tannins and Other Phenolics: We Need a Change in Approach. Funct. Ecol..

[49] Dobreva M.A., Green R.J., Mueller-Harvey I., Salminen J.-P., Howlin B.J., Frazier R.A. (2014). Size and Molecular Flexibility Affect the Binding of Ellagitannins to Bovine Serum Albumin. J. Agric. Food Chem..

[50] Karonen M., Oraviita M., Mueller-Harvey I., Salminen J.-P., Green R.J. (2015). Binding of an Oligomeric Ellagitannin Series to Bovine Serum Albumin (BSA): Analysis by Isothermal Titration Calorimetry (ITC). J. Agric. Food Chem..

[51] Karonen M., Parker J., Agrawal A., Salminen J.-P. (2010). First Evidence of Hexameric and Heptameric Ellagitannins in Plants Detected by Liquid Chromatography/Electrospray Ionisation Mass Spectrometry. Rapid Commun. Mass Spectrom..

[52] Baert N., Karonen M., Salminen J.-P. (2015). Isolation, Characterisation and Quantification of the Main Oligomeric Macrocyclic Ellagitannins in *Epilobium angustifolium* by Ultra-High Performance Chromatography with Diode Array Detection and Electrospray Tandem Mass Spectrometry. J. Chromatogr. A.

[53] Leppä M.M., Laitila J.E., Salminen J.-P. (2020). Distribution of Protein Precipitation Capacity within Variable Proanthocyanidin Fingerprints. Molecyles.

[54] Ossipov V., Salminen J.-P., Ossipova S., Haukioja E., Pihlaja K. (2003). Gallic Acid and Hydrolysable Tannins Are Formed in Birch Leaves from an Intermediate Compound of the Shikimate Pathway. Biochem. Syst. Ecol..

[55] James A.M., Ma D., Mellway R., Gesell A., Yoshida K., Walker V., Tran L., Stewart D., Reichelt M., Suvanto J. (2017). Poplar MYB115 and MYB134 Transcription Factors Regulate Proanthocyanidin Synthesis and Structure. Plant Physiol..

[56] Boege K., Marquis R.J. (2005). Facing Herbivory as You Grow up: The Ontogeny of Resistance in Plants. Trends Ecol. Evol..

[57] Slimestad R., Hostettmann K. (1996). Characterisation of Phenolic Constituents from Juvenile and Mature Needles of Norway Spruce by Means of High Performance Liquid Chromatography-Mass Spectrometry. Phytochem. Anal..

[58] Hagerman A.E. (1987). Radial Diffusion Method for Determining Tannin in Plant Extracts. J. Chem. Ecol..

[59] Abràmoff M.D., Magalhães P.J., Ram S.J. (2004). Image Processing with ImageJ. Biophotonics Int..

[60] Malisch C.S., Lüscher A., Baert N., Engström M.T., Studer B., Fryganas C., Suter D., Mueller-Harvey I., Salminen J.P. (2015). Large Variability of Proanthocyanidin Content and Composition in Sainfoin (*Onobrychis viciifolia*). J. Agric. Food Chem..

