# Supplementary material for: Mass Spectrometric Fingerprint Mapping Reveals Species-Specific Differences in Plant Polyphenols and Related Bioactivities
Source: Molecules. 2023 Aug 31;28(17):6388. doi: 10.3390/molecules28176388 (PMC10490256; doi:10.3390/molecules28176388)
Supplement: Supplementary file 1 [file molecules-28-06388-s001.zip › molecules-2454233-supplementary.pdf]

Supplementary material

Vanhakylä S. & Salminen J.-P. (2023) Mass Spectrometric Fingerprint Mapping Reveals Species-Specific Differences in Plant Polyphenols and Related Bioactivities

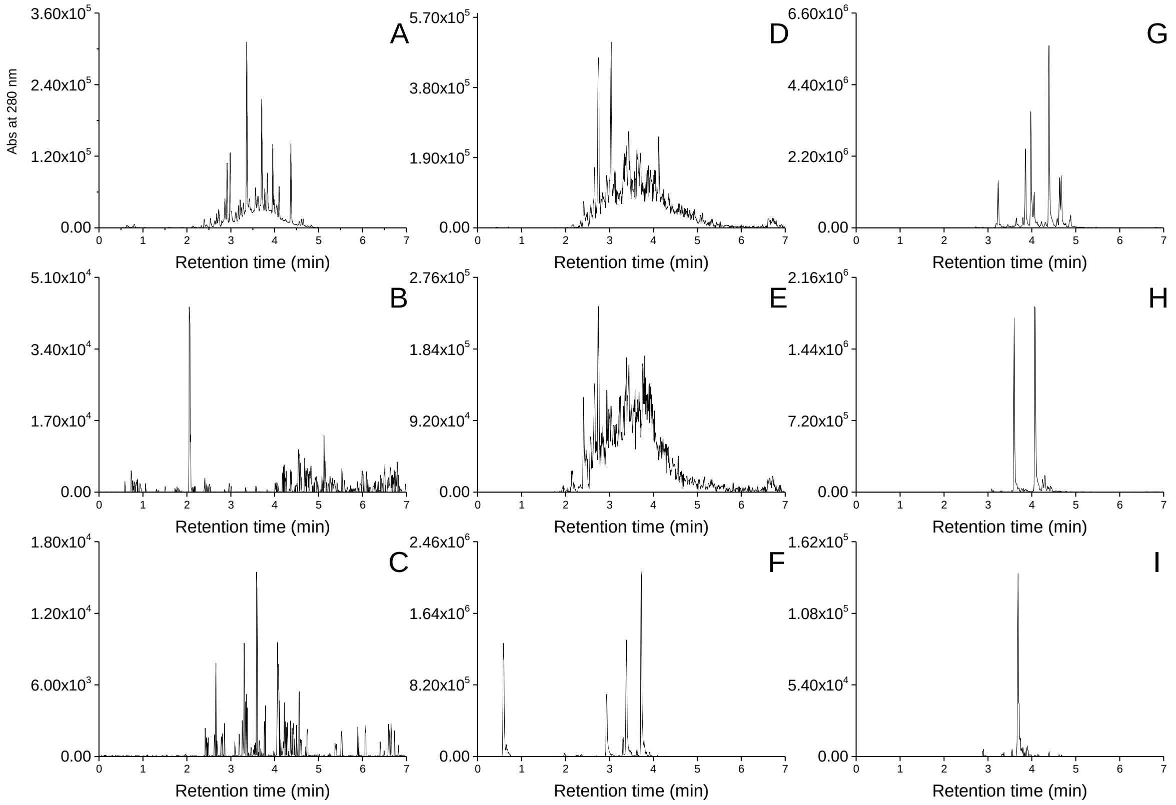

Figure S1. Examples of UHPLC-UV and group-specific UHPLC-MS/MS fingerprints recorded from the polyphenol extract of *Gymnocarpium dryopteris*. (A) UV traces at 280 nm, (B) galloyl derivative fingerprint, (C) hexahydroxydiphenoyl derivative fingerprint, (D) procyanidin polymer fingerprint, (E) prodelphinidin polymer fingerprint, (F) quinic acid derivative fingerprint (the peak in at 0.8 min is free quinic acid found in plants, i.e. it is not a polyphenol), (G) kaempferol derivative fingerprint, (H) quercetin derivative fingerprint and (I) myricetin derivative fingerprint. The y-axes are scaled to the most intensive peak of each fingerprint.

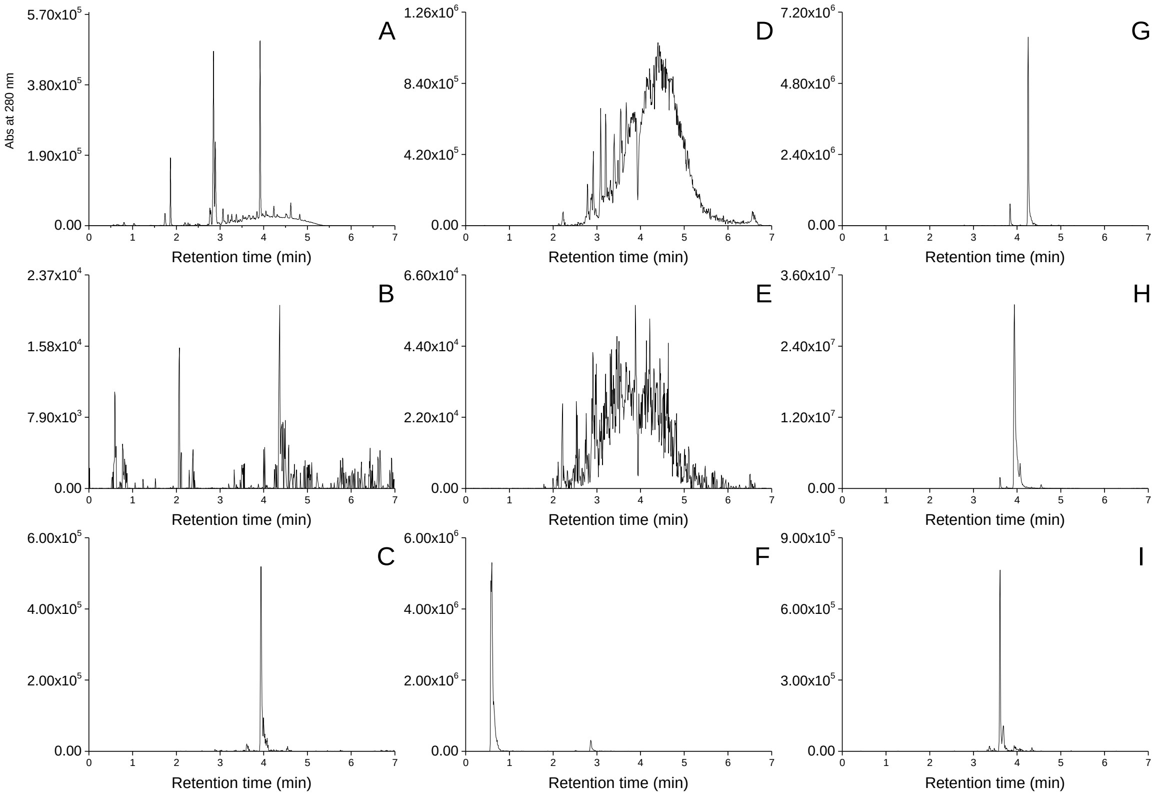

Figure S2. Examples of UHPLC-UV and group-specific UHPLC-MS/MS fingerprints recorded from the polyphenol extract of *Juniperus communis*. (A) UV traces at 280 nm, (B) galloyl derivative fingerprint, (C) hexahydroxydiphenoyl derivative fingerprint, (D) procyanidin polymer fingerprint, (E) prodelphinidin polymer fingerprint, (F) quinic acid derivative fingerprint (the peak in at 0.8 min is free quinic acid found in plants, i.e. it is not a polyphenol), (G) kaempferol derivative fingerprint, (H) quercetin derivative fingerprint and (I) myricetin derivative fingerprint. The y-axes are scaled to the most intensive peak of each fingerprint.

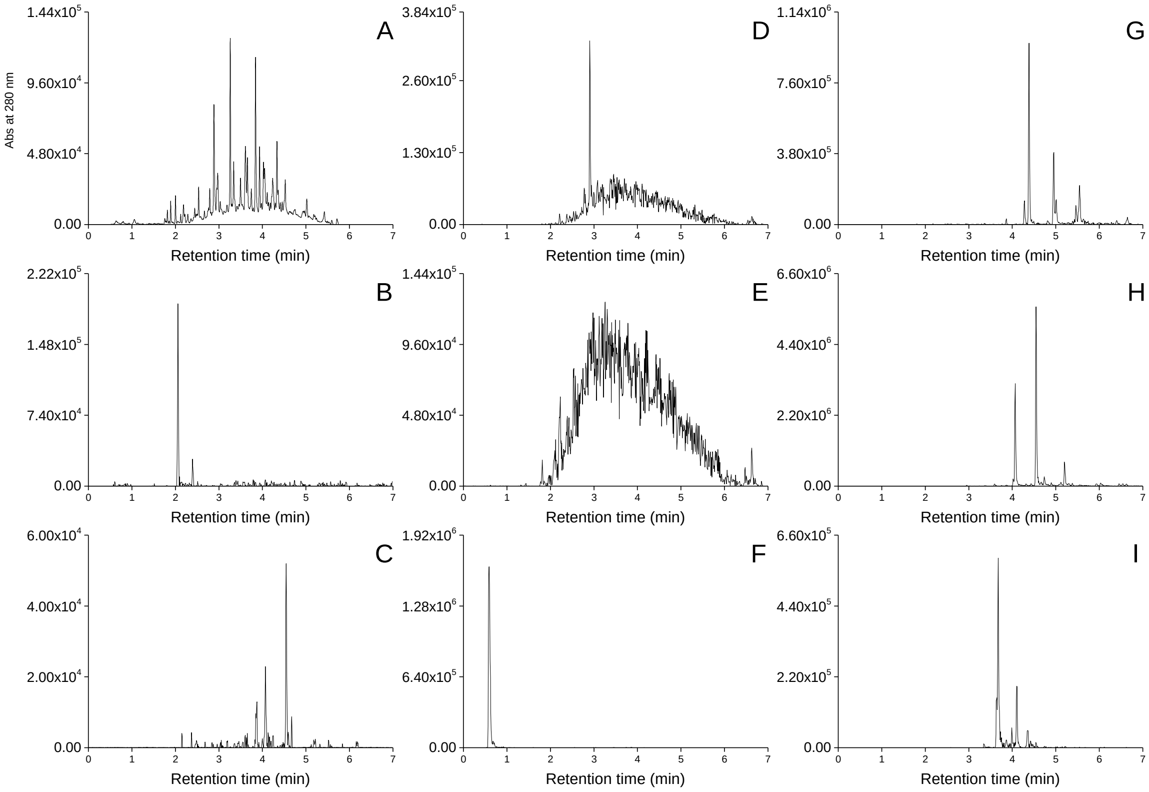

Figure S3. Examples of UHPLC-UV and group-specific UHPLC-MS/MS fingerprints recorded from the polyphenol extract of *Pinus sylvestris*. (A) UV traces at 280 nm, (B) galloyl derivative fingerprint, (C) hexahydroxydiphenoyl derivative fingerprint, (D) procyanidin polymer fingerprint, (E) prodelphinidin polymer fingerprint, (F) quinic acid derivative fingerprint (the peak in at 0.8 min is free quinic acid found in plants, i.e. it is not a polyphenol), (G) kaempferol derivative fingerprint, (H) quercetin derivative fingerprint and (I) myricetin derivative fingerprint. The y-axes are scaled to the most intensive peak of each fingerprint.

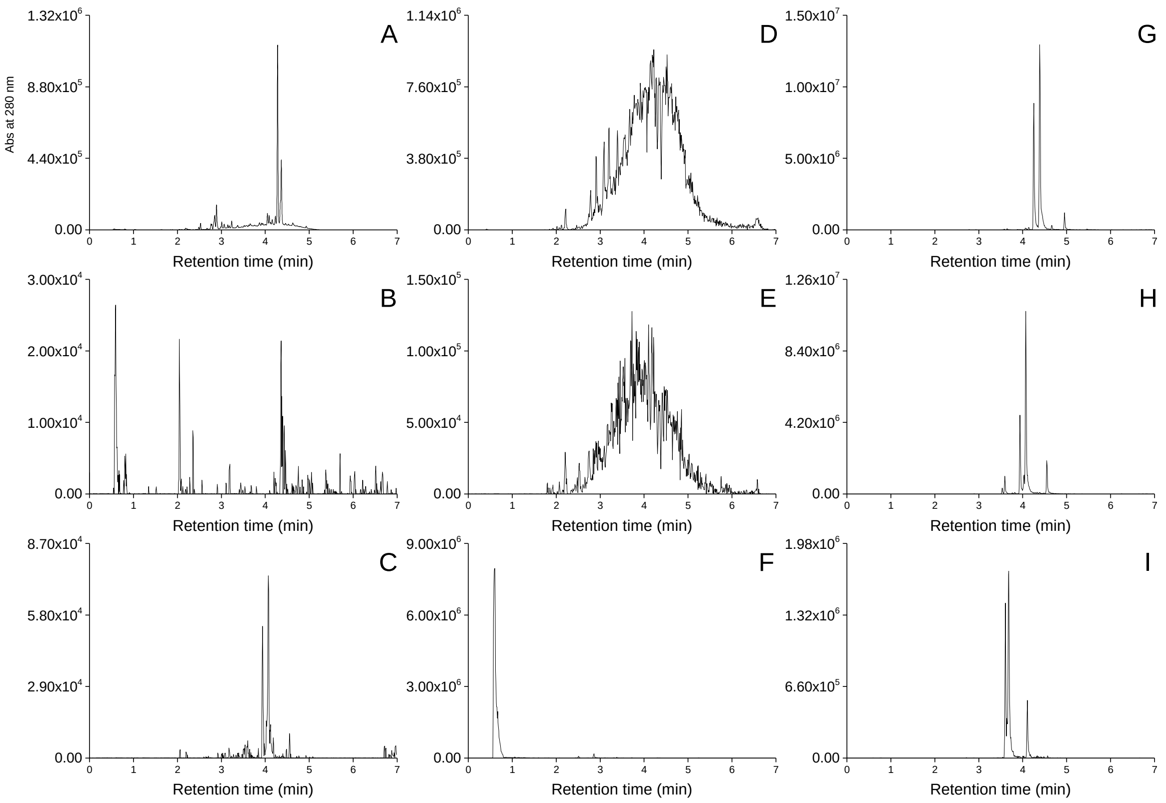

Figure S4. Examples of UHPLC-UV and group-specific UHPLC-MS/MS fingerprints recorded from the polyphenol extract of *Picea abies*. (A) UV traces at 280 nm, (B) galloyl derivative fingerprint, (C) hexahydroxydiphenoyl derivative fingerprint, (D) procyanidin polymer fingerprint, (E) prodelphinidin polymer fingerprint, (F) quinic acid derivative fingerprint (the peak in at 0.8 min is free quinic acid found in plants, i.e. it is not a polyphenol), (G) kaempferol derivative fingerprint, (H) quercetin derivative fingerprint and (I) myricetin derivative fingerprint. The y-axes are scaled to the most intensive peak of each fingerprint.

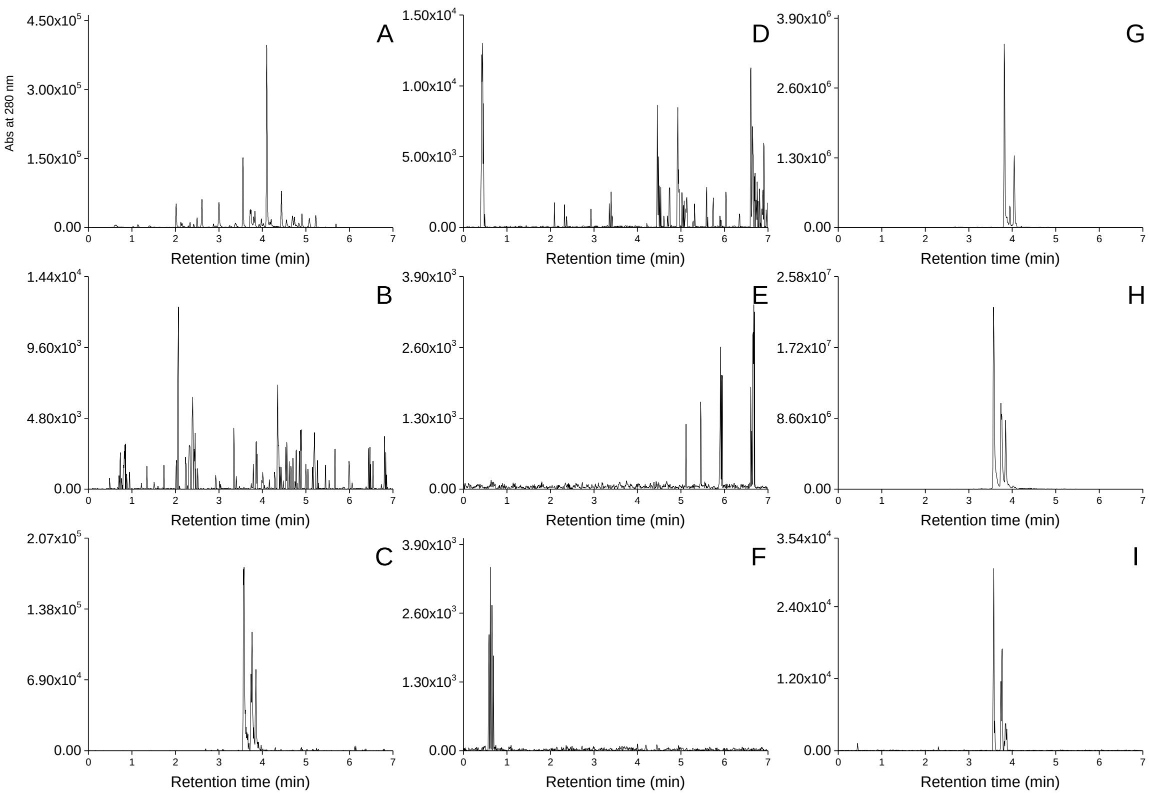

Figure S5. Examples of UHPLC-UV and group-specific UHPLC-MS/MS fingerprints recorded from the polyphenol extract of *Caltha palustris*. (A) UV traces at 280 nm, (B) galloyl derivative fingerprint, (C) hexahydroxydiphenoyl derivative fingerprint, (D) procyanidin polymer fingerprint, (E) prodelphinidin polymer fingerprint, (F) quinic acid derivative fingerprint, (G) kaempferol derivative fingerprint (the peak in at 0.8 min is free quinic acid found in plants, i.e. it is not a polyphenol), (H) quercetin derivative fingerprint and (I) myricetin derivative fingerprint. The y-axes are scaled to the most intensive peak of each fingerprint.

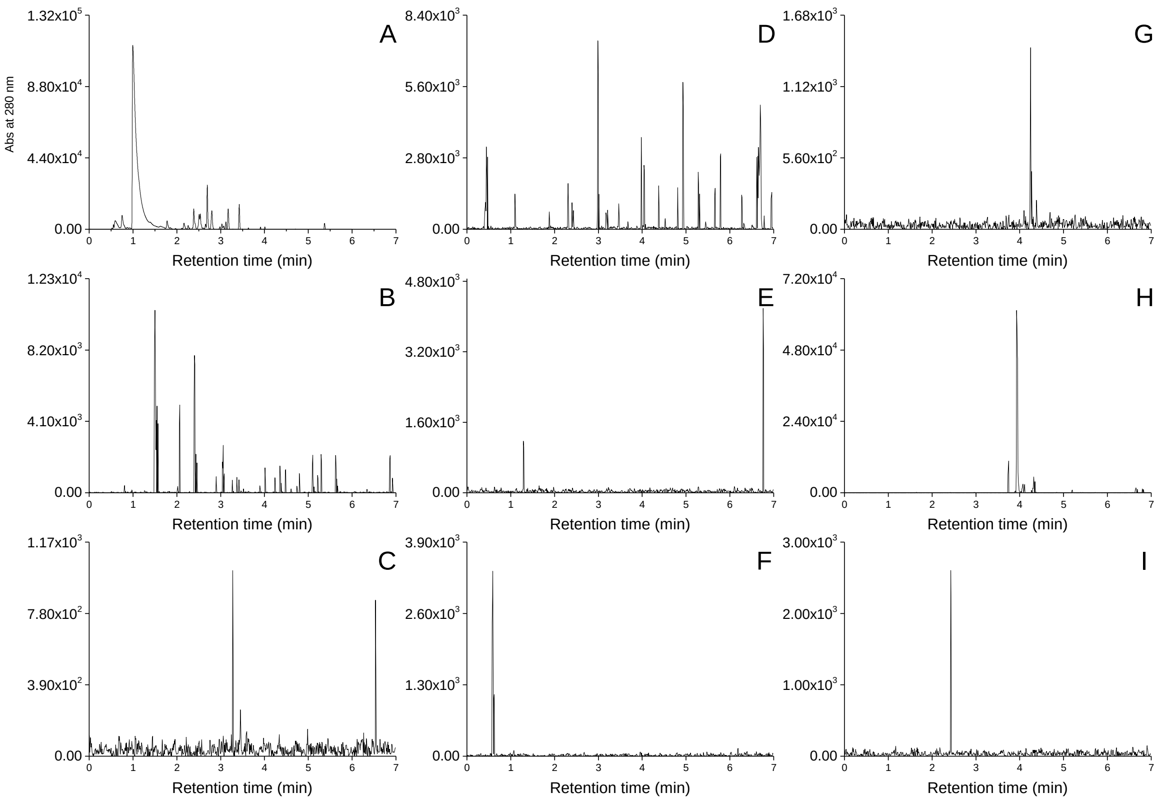

Figure S6. Examples of UHPLC-UV and group-specific UHPLC-MS/MS fingerprints recorded from the polyphenol extract of *Chelidonium majus*. (A) UV traces at 280 nm, (B) galloyl derivative fingerprint, (C) hexahydroxydiphenoyl derivative fingerprint, (D) procyanidin polymer fingerprint, (E) prodelphinidin polymer fingerprint, (F) quinic acid derivative fingerprint (the peak in at 0.8 min is free quinic acid found in plants, i.e. it is not a polyphenol), (G) kaempferol derivative fingerprint, (H) quercetin derivative fingerprint and (I) myricetin derivative fingerprint. The y-axes are scaled to the most intensive peak of each fingerprint.

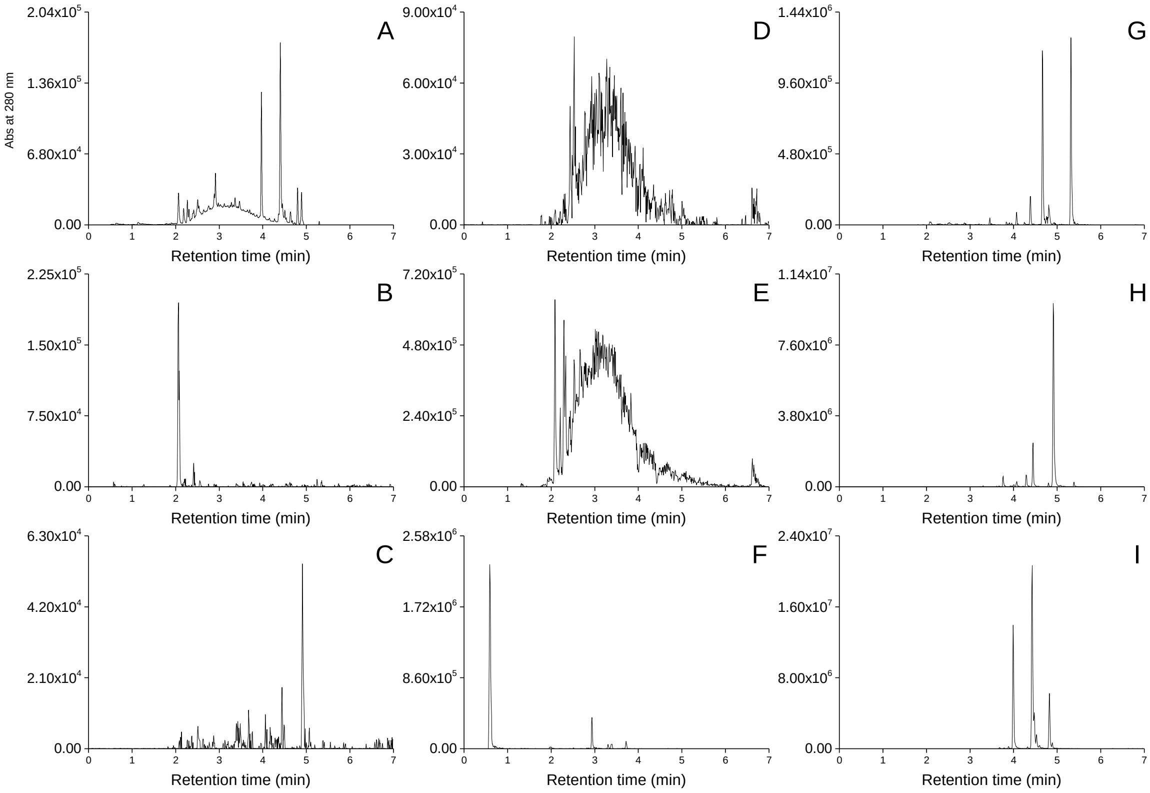

Figure S7. Examples of UHPLC-UV and group-specific UHPLC-MS/MS fingerprints recorded from the polyphenol extract of *Ribes alpinum*. (A) UV traces at 280 nm, (B) galloyl derivative fingerprint, (C) hexahydroxydiphenoyl derivative fingerprint, (D) procyanidin polymer fingerprint, (E) prodelphinidin polymer fingerprint, (F) quinic acid derivative fingerprint (the peak in at 0.8 min is free quinic acid found in plants, i.e. it is not a polyphenol), (G) kaempferol derivative fingerprint, (H) quercetin derivative fingerprint and (I) myricetin derivative fingerprint. The y-axes are scaled to the most intensive peak of each fingerprint.

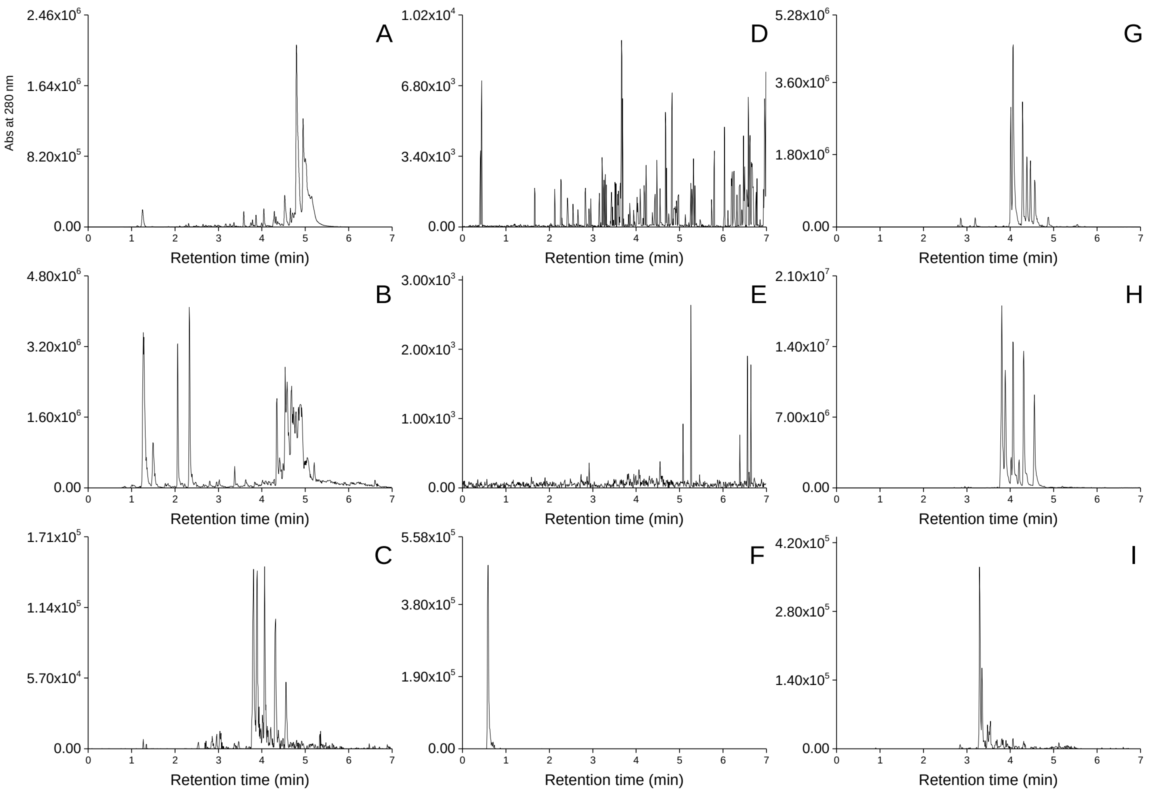

Figure S8. Examples of UHPLC-UV and group-specific UHPLC-MS/MS fingerprints recorded from the polyphenol extract of *Paeonia lactiflora*. (A) UV traces at 280 nm, (B) galloyl derivative fingerprint, (C) hexahydroxydiphenoyl derivative fingerprint, (D) procyanidin polymer fingerprint, (E) prodelphinidin polymer fingerprint, (F) quinic acid derivative fingerprint (the peak in at 0.8 min is free quinic acid found in plants, i.e. it is not a polyphenol), (G) kaempferol derivative fingerprint, (H) quercetin derivative fingerprint and (I) myricetin derivative fingerprint. The y-axes are scaled to the most intensive peak of each fingerprint.

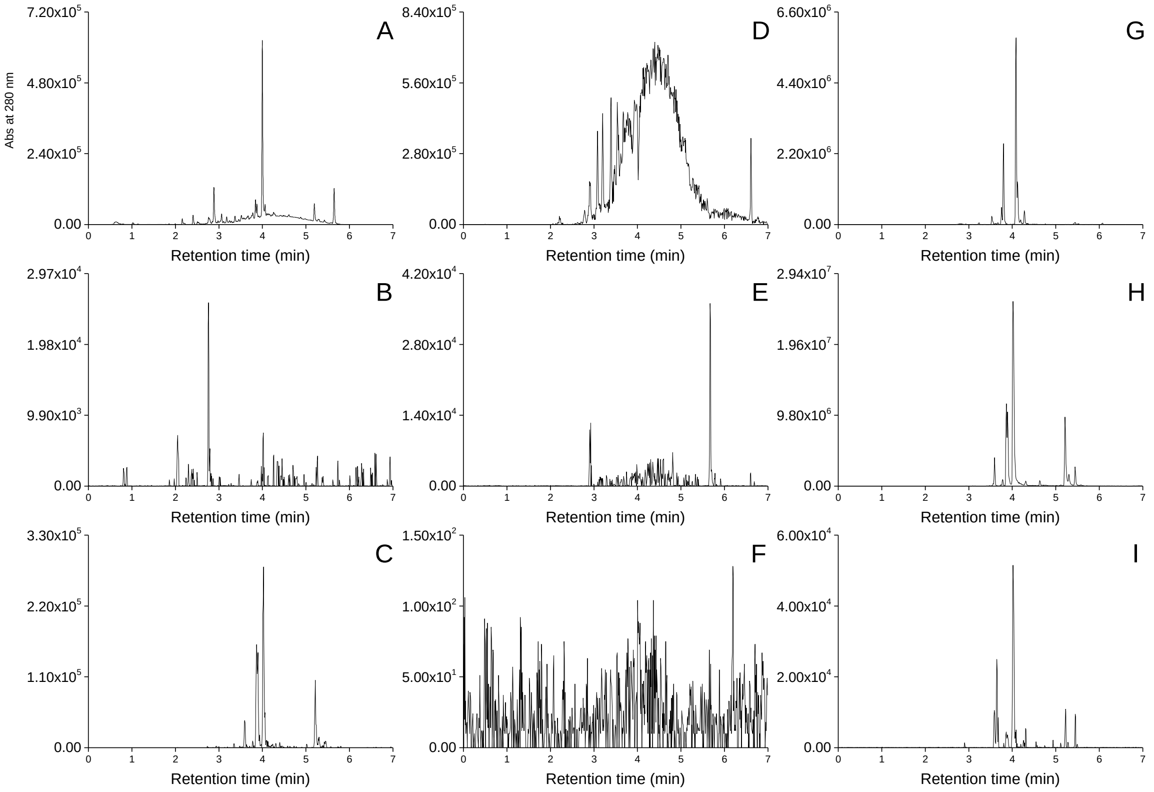

Figure S9. Examples of UHPLC-UV and group-specific UHPLC-MS/MS fingerprints recorded from the polyphenol extract of *Trifolium hybridum*. (A) UV traces at 280 nm, (B) galloyl derivative fingerprint, (C) hexahydroxydiphenoyl derivative fingerprint, (D) procyanidin polymer fingerprint, (E) prodelphinidin polymer fingerprint, (F) quinic acid derivative fingerprint (the peak in at 0.8 min is free quinic acid found in plants, i.e. it is not a polyphenol), (G) kaempferol derivative fingerprint, (H) quercetin derivative fingerprint and (I) myricetin derivative fingerprint. The y-axes are scaled to the most intensive peak of each fingerprint.

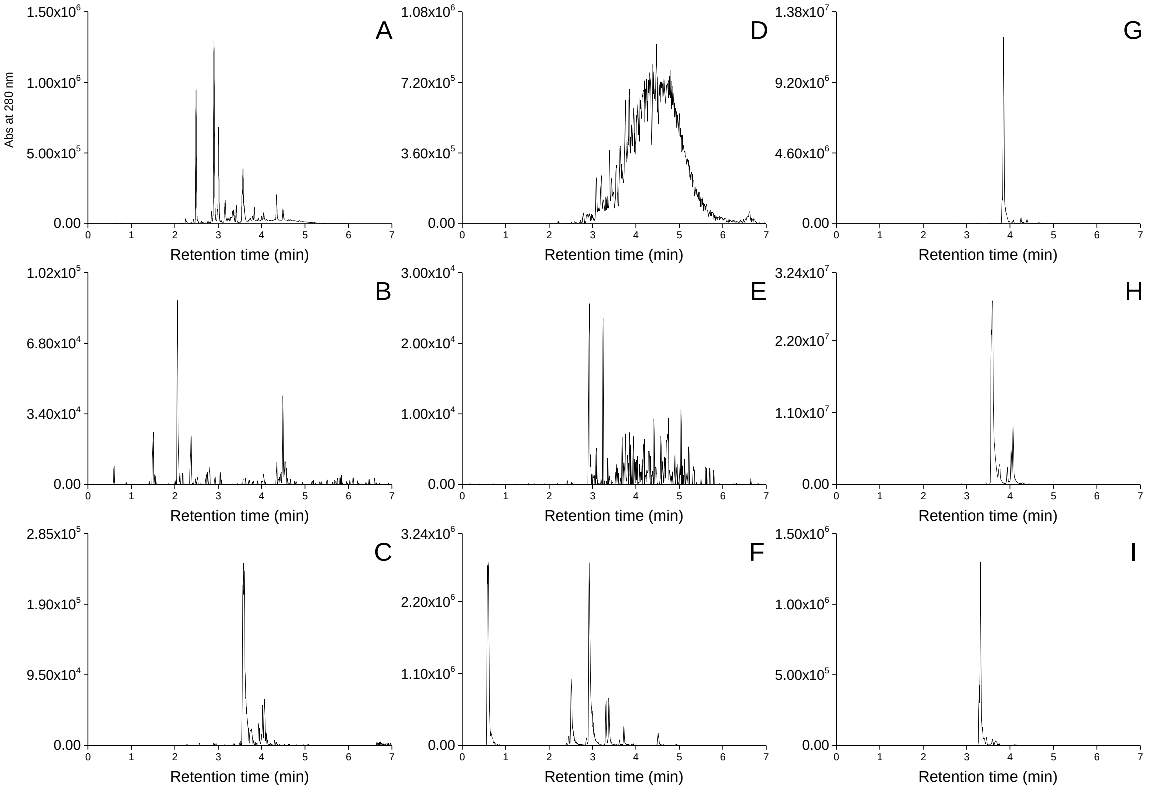

Figure S10. Examples of UHPLC-UV and group-specific UHPLC-MS/MS fingerprints recorded from the polyphenol extract of *Sorbus aucuparia*. (A) UV traces at 280 nm, (B) galloyl derivative fingerprint, (C) hexahydroxydiphenoyl derivative fingerprint, (D) procyanidin polymer fingerprint, (E) prodelphinidin polymer fingerprint, (F) quinic acid derivative fingerprint (the peak in at 0.8 min is free quinic acid found in plants, i.e. it is not a polyphenol), (G) kaempferol derivative fingerprint, (H) quercetin derivative fingerprint and (I) myricetin derivative fingerprint. The y-axes are scaled to the most intensive peak of each fingerprint.

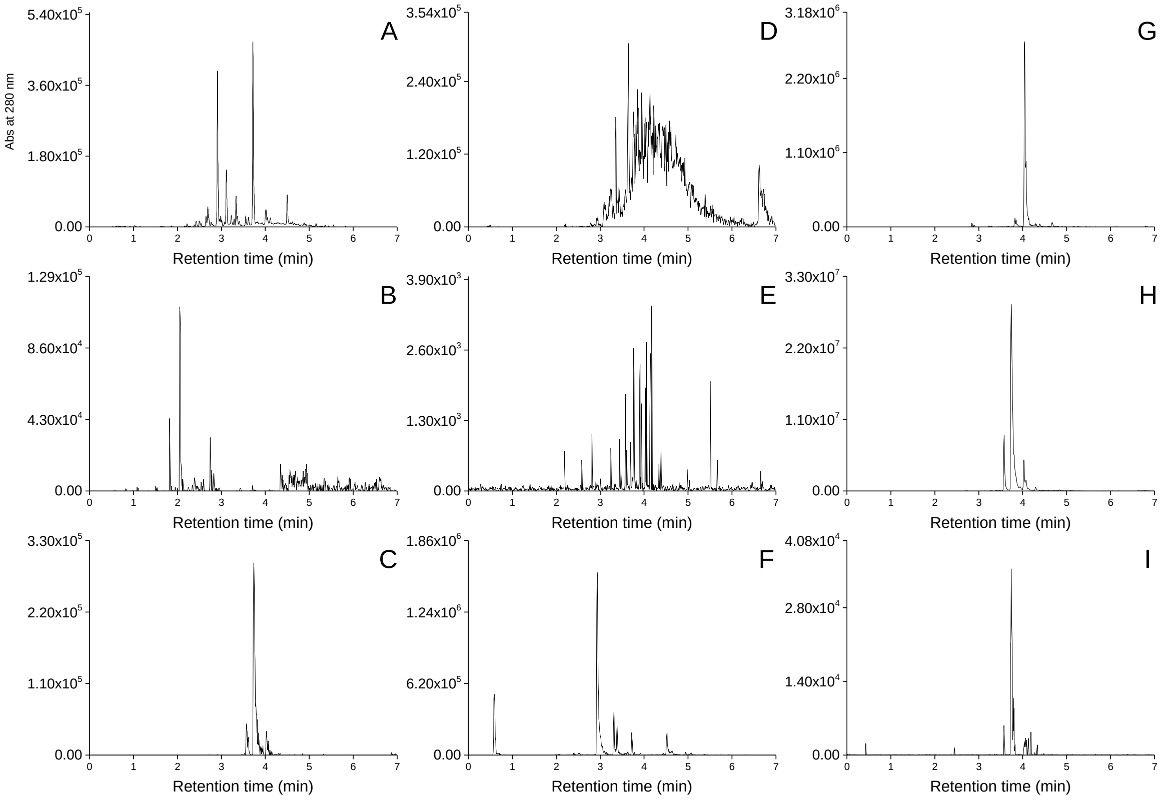

Figure S11. Examples of UHPLC-UV and group-specific UHPLC-MS/MS fingerprints recorded from the polyphenol extract of *Prunus padus*. (A) UV traces at 280 nm, (B) galloyl derivative fingerprint, (C) hexahydroxydiphenoyl derivative fingerprint, (D) procyanidin polymer fingerprint, (E) prodelphinidin polymer fingerprint, (F) quinic acid derivative fingerprint (the peak in at 0.8 min is free quinic acid found in plants, i.e. it is not a polyphenol), (G) kaempferol derivative fingerprint, (H) quercetin derivative fingerprint and (I) myricetin derivative fingerprint. The y-axes are scaled to the most intensive peak of each fingerprint.

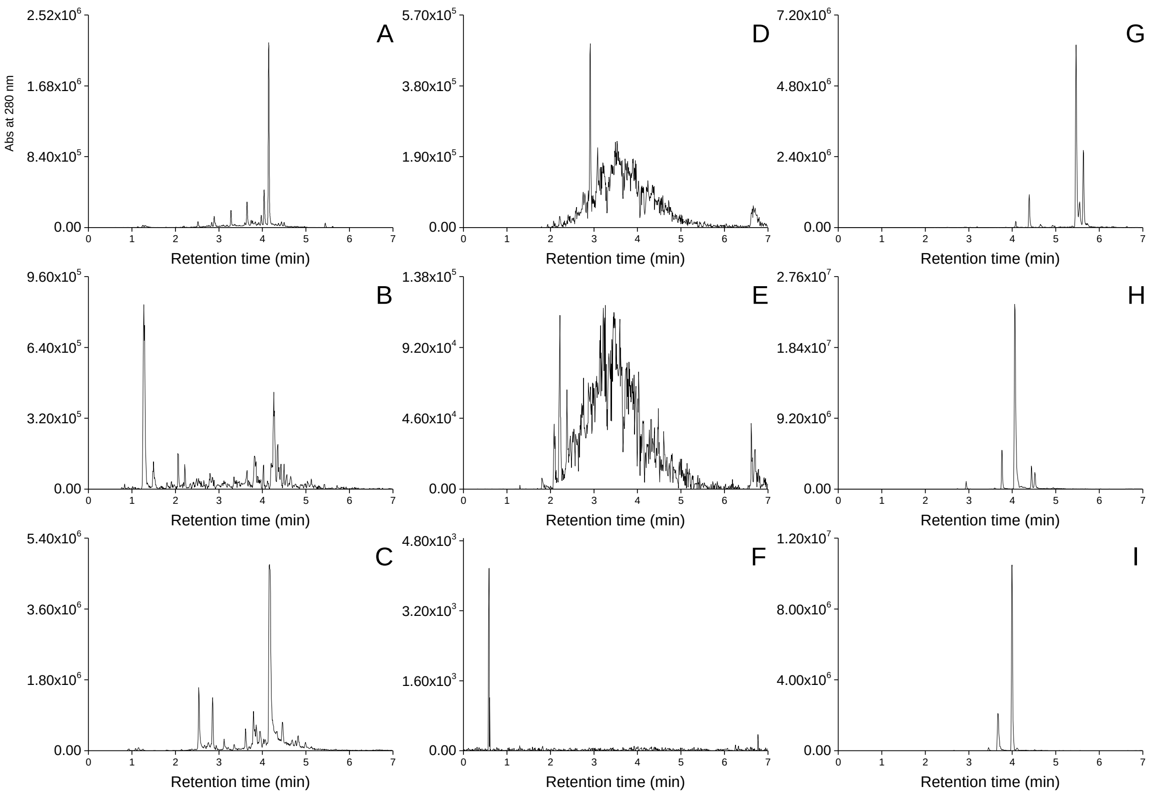

Figure S12. Examples of UHPLC-UV and group-specific UHPLC-MS/MS fingerprints recorded from the polyphenol extract of *Argentina anserina*. (A) UV traces at 280 nm, (B) galloyl derivative fingerprint, (C) hexahydroxydiphenoyl derivative fingerprint, (D) procyanidin polymer fingerprint, (E) prodelphinidin polymer fingerprint, (F) quinic acid derivative fingerprint (the peak in at 0.8 min is free quinic acid found in plants, i.e. it is not a polyphenol), (G) kaempferol derivative fingerprint, (H) quercetin derivative fingerprint and (I) myricetin derivative fingerprint. The y-axes are scaled to the most intensive peak of each fingerprint.

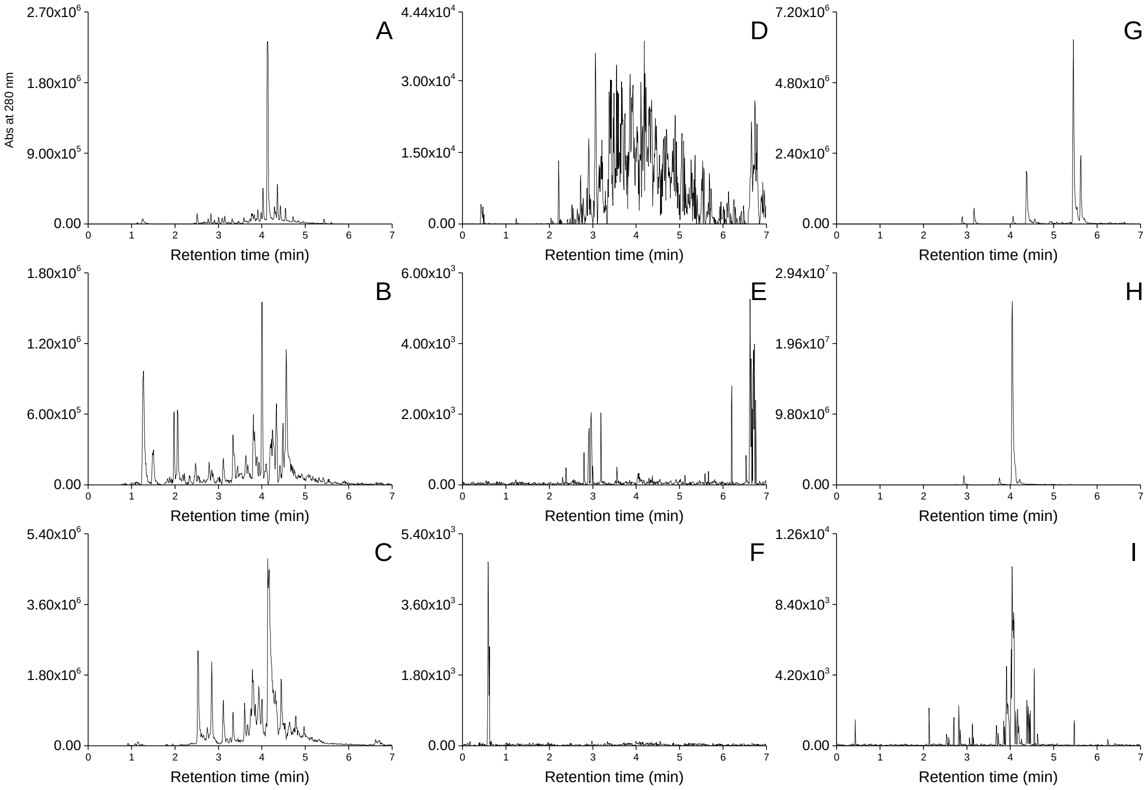

Figure S13. Examples of UHPLC-UV and group-specific UHPLC-MS/MS fingerprints recorded from the polyphenol extract of *Comarum palustre*. (A) UV traces at 280 nm, (B) galloyl derivative fingerprint, (C) hexahydroxydiphenoyl derivative fingerprint, (D) procyanidin polymer fingerprint, (E) prodelphinidin polymer fingerprint, (F) quinic acid derivative fingerprint (the peak in at 0.8 min is free quinic acid found in plants, i.e. it is not a polyphenol), (G) kaempferol derivative fingerprint, (H) quercetin derivative fingerprint and (I) myricetin derivative fingerprint. The y-axes are scaled to the most intensive peak of each fingerprint.

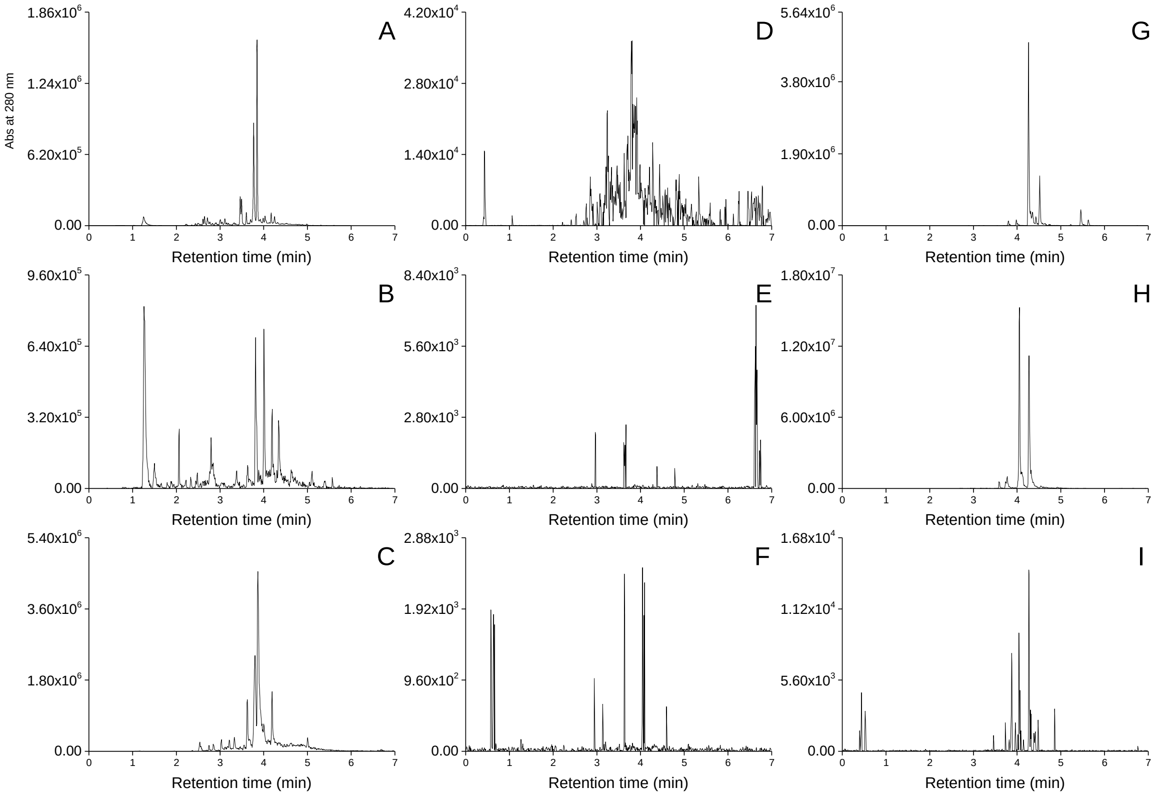

Figure S14. Examples of UHPLC-UV and group-specific UHPLC-MS/MS fingerprints recorded from the polyphenol extract of *Rubus saxatilis*. (A) UV traces at 280 nm, (B) galloyl derivative fingerprint, (C) hexahydroxydiphenoyl derivative fingerprint, (D) procyanidin polymer fingerprint, (E) prodelphinidin polymer fingerprint, (F) quinic acid derivative fingerprint (the peak in at 0.8 min is free quinic acid found in plants, i.e. it is not a polyphenol), (G) kaempferol derivative fingerprint, (H) quercetin derivative fingerprint and (I) myricetin derivative fingerprint. The y-axes are scaled to the most intensive peak of each fingerprint.

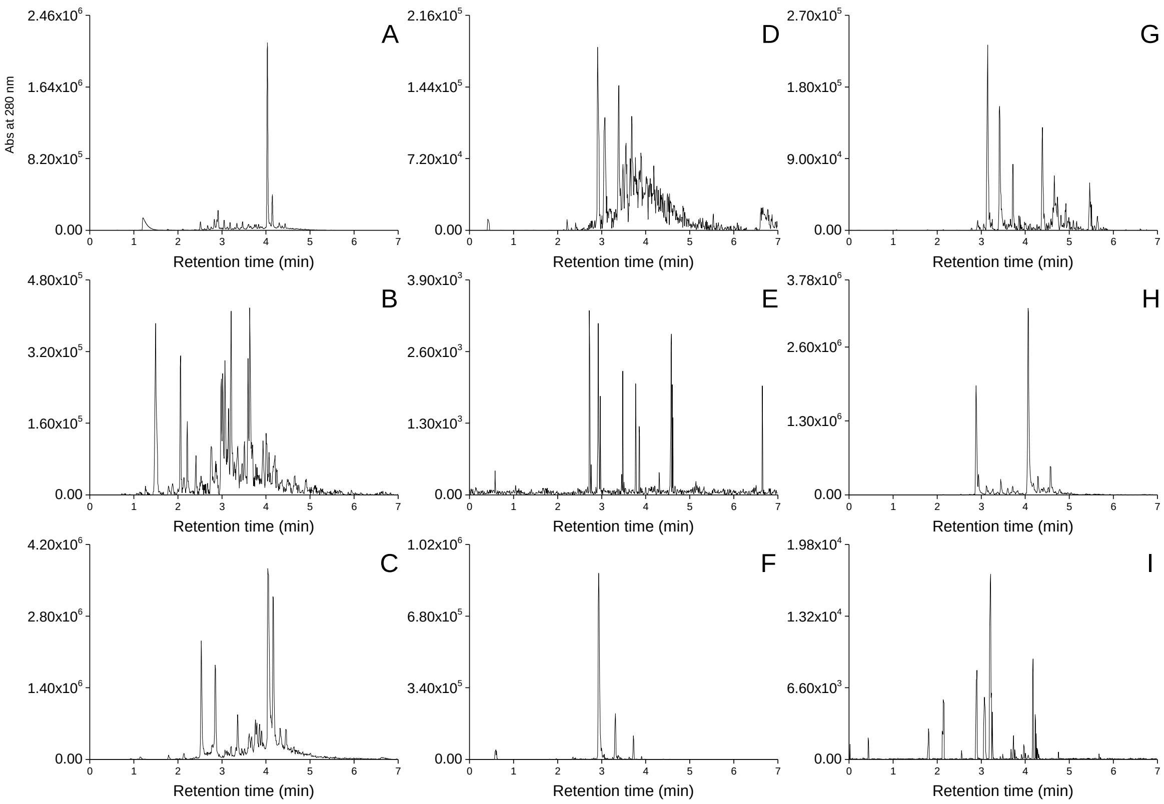

Figure S15. Examples of UHPLC-UV and group-specific UHPLC-MS/MS fingerprints recorded from the polyphenol extract of *Geum rivale*. (A) UV traces at 280 nm, (B) galloyl derivative fingerprint, (C) hexahydroxydiphenoyl derivative fingerprint, (D) procyanidin polymer fingerprint, (E) prodelphinidin polymer fingerprint, (F) quinic acid derivative fingerprint (the peak in at 0.8 min is free quinic acid found in plants, i.e. it is not a polyphenol), (G) kaempferol derivative fingerprint, (H) quercetin derivative fingerprint and (I) myricetin derivative fingerprint. The y-axes are scaled to the most intensive peak of each fingerprint.

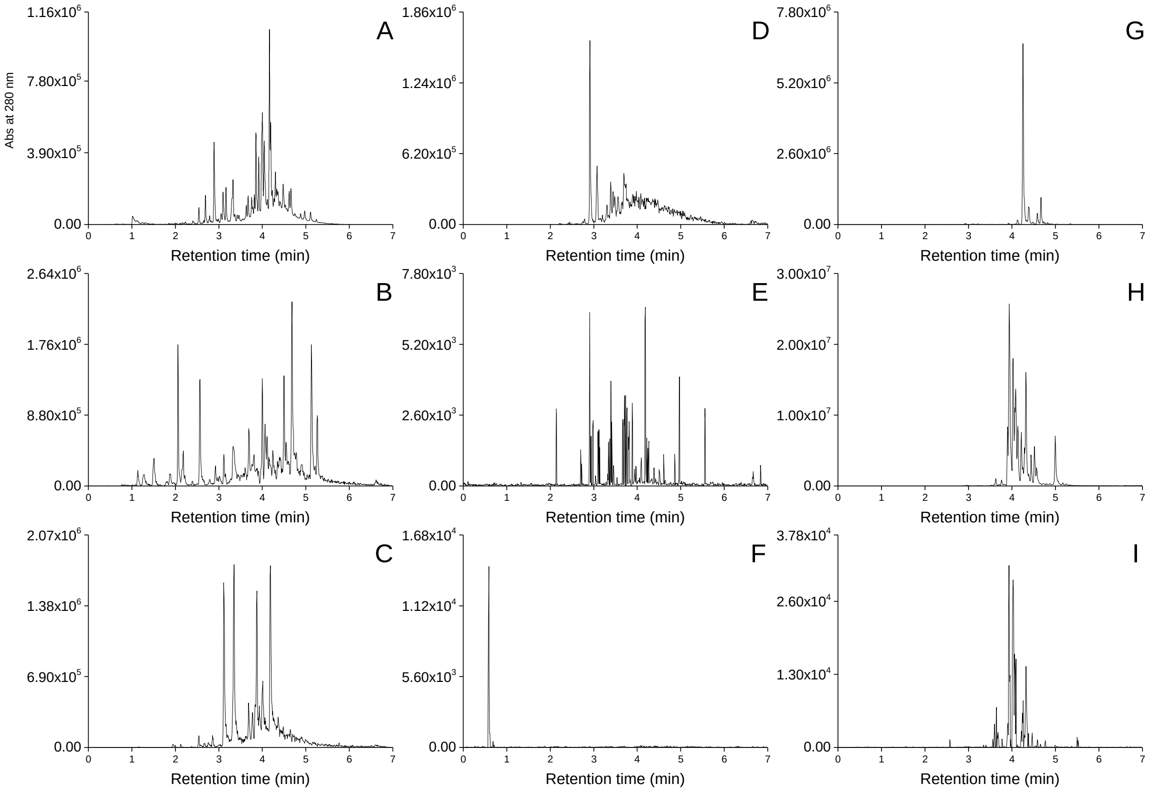

Figure S16. Examples of UHPLC-UV and group-specific UHPLC-MS/MS fingerprints recorded from the polyphenol extract of *Filipendula ulmaria*. (A) UV traces at 280 nm, (B) galloyl derivative fingerprint, (C) hexahydroxydiphenoyl derivative fingerprint, (D) procyanidin polymer fingerprint, (E) prodelphinidin polymer fingerprint, (F) quinic acid derivative fingerprint (the peak in at 0.8 min is free quinic acid found in plants, i.e. it is not a polyphenol), (G) kaempferol derivative fingerprint, (H) quercetin derivative fingerprint and (I) myricetin derivative fingerprint. The y-axes are scaled to the most intensive peak of each fingerprint.

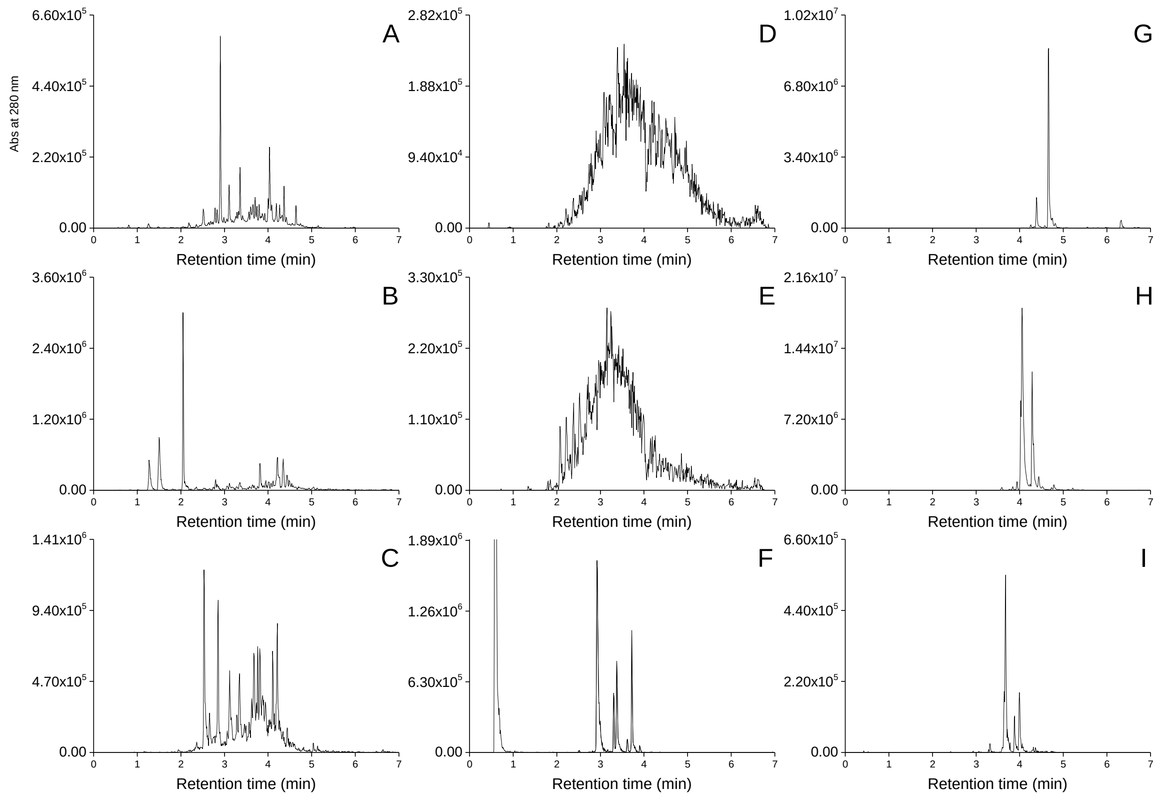

Figure S17. Examples of UHPLC-UV and group-specific UHPLC-MS/MS fingerprints recorded from the polyphenol extract of *Betula pubescens*. (A) UV traces at 280 nm, (B) galloyl derivative fingerprint, (C) hexahydroxydiphenoyl derivative fingerprint, (D) procyanidin polymer fingerprint, (E) prodelphinidin polymer fingerprint, (F) quinic acid derivative fingerprint (the peak in at 0.8 min is free quinic acid found in plants, i.e. it is not a polyphenol), (G) kaempferol derivative fingerprint, (H) quercetin derivative fingerprint and (I) myricetin derivative fingerprint. The y-axes are scaled to the most intensive peak of each fingerprint.

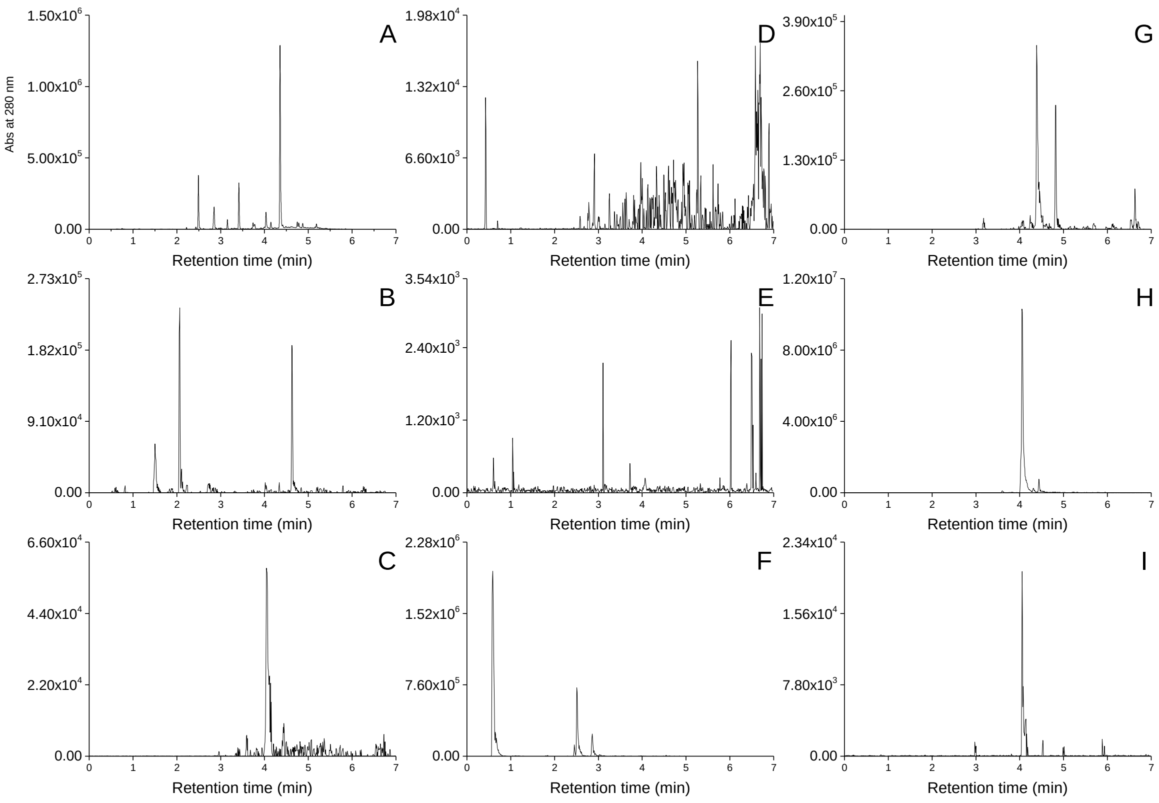

Figure S18. Examples of UHPLC-UV and group-specific UHPLC-MS/MS fingerprints recorded from the polyphenol extract of *Alnus glutinosa*. (A) UV traces at 280 nm, (B) galloyl derivative fingerprint, (C) hexahydroxydiphenoyl derivative fingerprint, (D) procyanidin polymer fingerprint, (E) prodelphinidin polymer fingerprint, (F) quinic acid derivative fingerprint (the peak in at 0.8 min is free quinic acid found in plants, i.e. it is not a polyphenol), (G) kaempferol derivative fingerprint, (H) quercetin derivative fingerprint and (I) myricetin derivative fingerprint. The y-axes are scaled to the most intensive peak of each fingerprint.

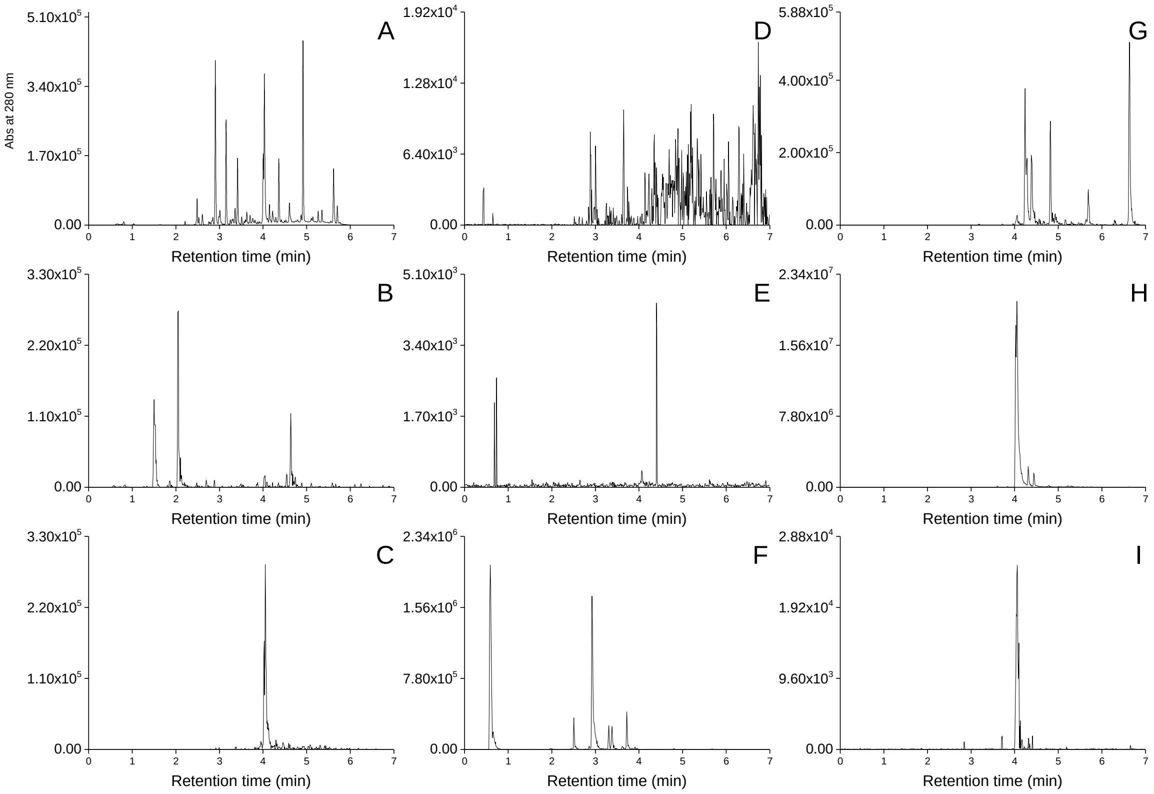

Figure S19. Examples of UHPLC-UV and group-specific UHPLC-MS/MS fingerprints recorded from the polyphenol extract of *Alnus incana*. (A) UV traces at 280 nm, (B) galloyl derivative fingerprint, (C) hexahydroxydiphenoyl derivative fingerprint, (D) procyanidin polymer fingerprint, (E) prodelphinidin polymer fingerprint, (F) quinic acid derivative fingerprint (the peak in at 0.8 min is free quinic acid found in plants, i.e. it is not a polyphenol), (G) kaempferol derivative fingerprint, (H) quercetin derivative fingerprint and (I) myricetin derivative fingerprint. The y-axes are scaled to the most intensive peak of each fingerprint.

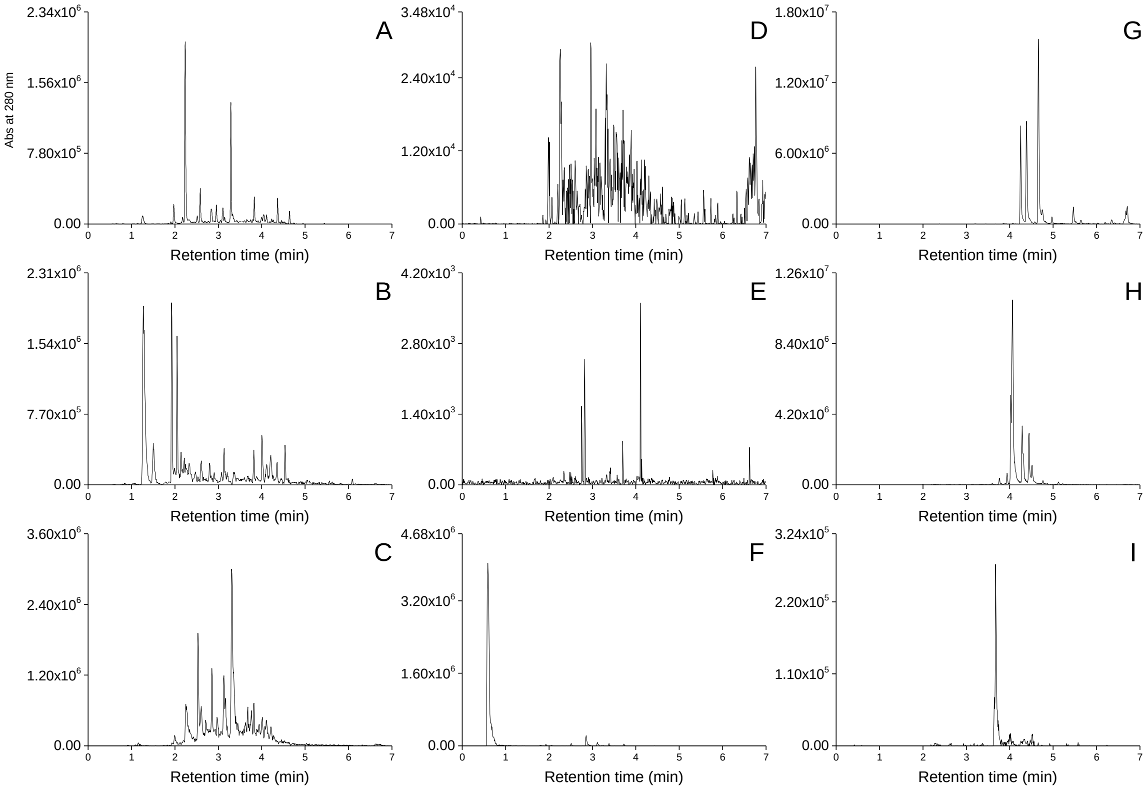

Figure S20. Examples of UHPLC-UV and group-specific UHPLC-MS/MS fingerprints recorded from the polyphenol extract of *Quercus robur*. (A) UV traces at 280 nm, (B) galloyl derivative fingerprint, (C) hexahydroxydiphenoyl derivative fingerprint, (D) procyanidin polymer fingerprint, (E) prodelphinidin polymer fingerprint, (F) quinic acid derivative fingerprint (the peak in at 0.8 min is free quinic acid found in plants, i.e. it is not a polyphenol), (G) kaempferol derivative fingerprint, (H) quercetin derivative fingerprint and (I) myricetin derivative fingerprint. The y-axes are scaled to the most intensive peak of each fingerprint.

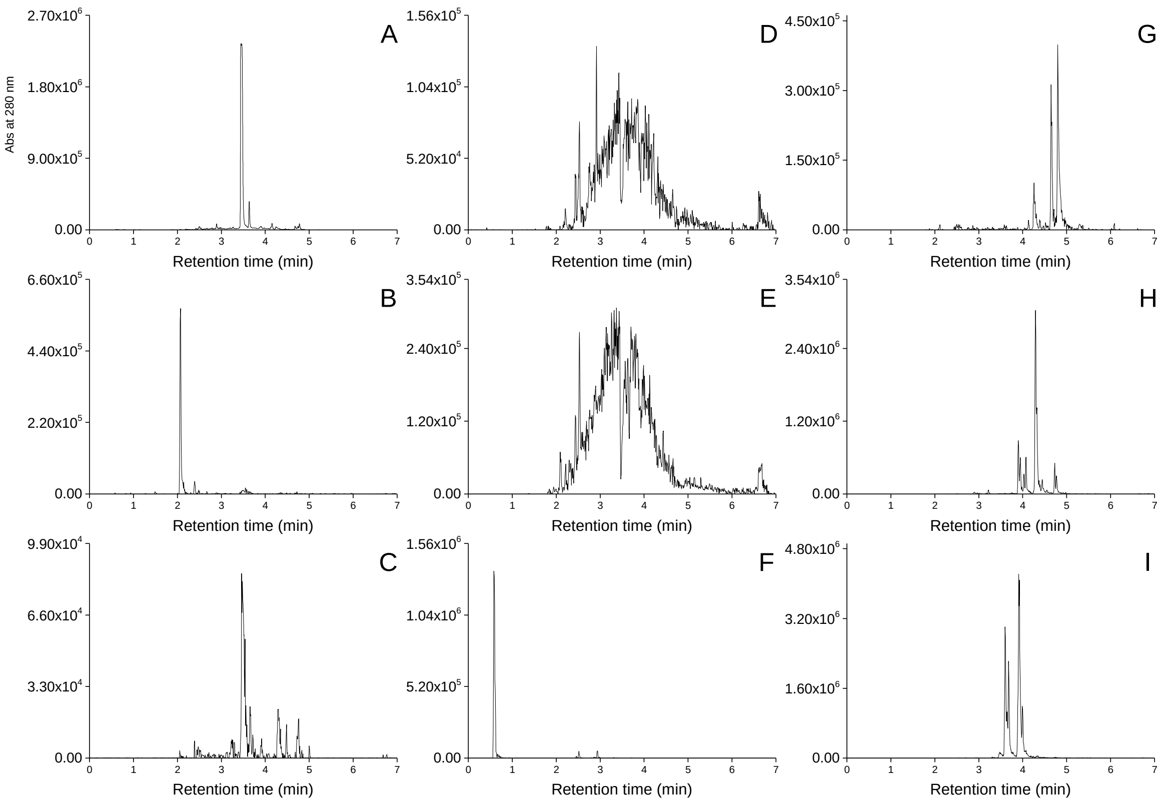

Figure S21. Examples of UHPLC-UV and group-specific UHPLC-MS/MS fingerprints recorded from the polyphenol extract of *Salix phylicifolia*. (A) UV traces at 280 nm, (B) galloyl derivative fingerprint, (C) hexahydroxydiphenoyl derivative fingerprint, (D) procyanidin polymer fingerprint, (E) prodelphinidin polymer fingerprint, (F) quinic acid derivative fingerprint (the peak in at 0.8 min is free quinic acid found in plants, i.e. it is not a polyphenol), (G) kaempferol derivative fingerprint, (H) quercetin derivative fingerprint and (I) myricetin derivative fingerprint. The y-axes are scaled to the most intensive peak of each fingerprint.

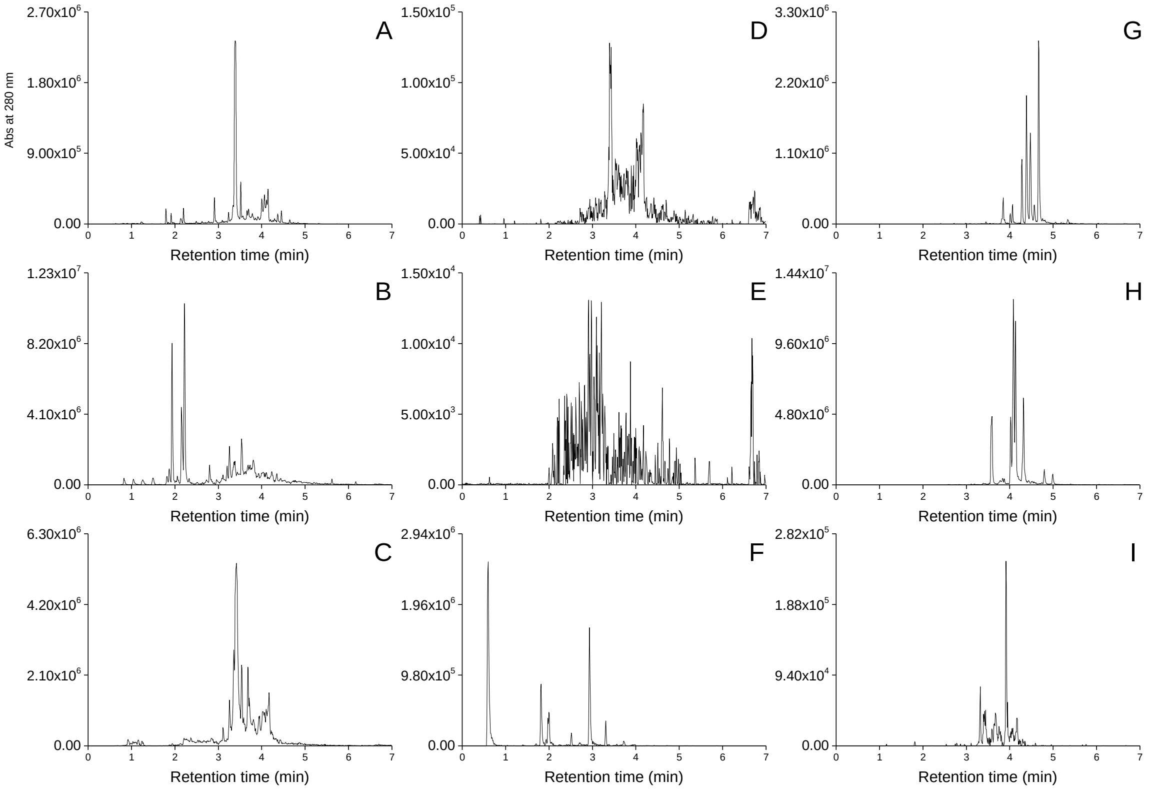

Figure S22. Examples of UHPLC-UV and group-specific UHPLC-MS/MS fingerprints recorded from the polyphenol extract of *Geranium sylvaticum*. (A) UV traces at 280 nm, (B) galloyl derivative fingerprint, (C) hexahydroxydiphenoyl derivative fingerprint, (D) procyanidin polymer fingerprint, (E) prodelphinidin polymer fingerprint, (F) quinic acid derivative fingerprint (the peak in at 0.8 min is free quinic acid found in plants, i.e. it is not a polyphenol), (G) kaempferol derivative fingerprint, (H) quercetin derivative fingerprint and (I) myricetin derivative fingerprint. The y-axes are scaled to the most intensive peak of each fingerprint.

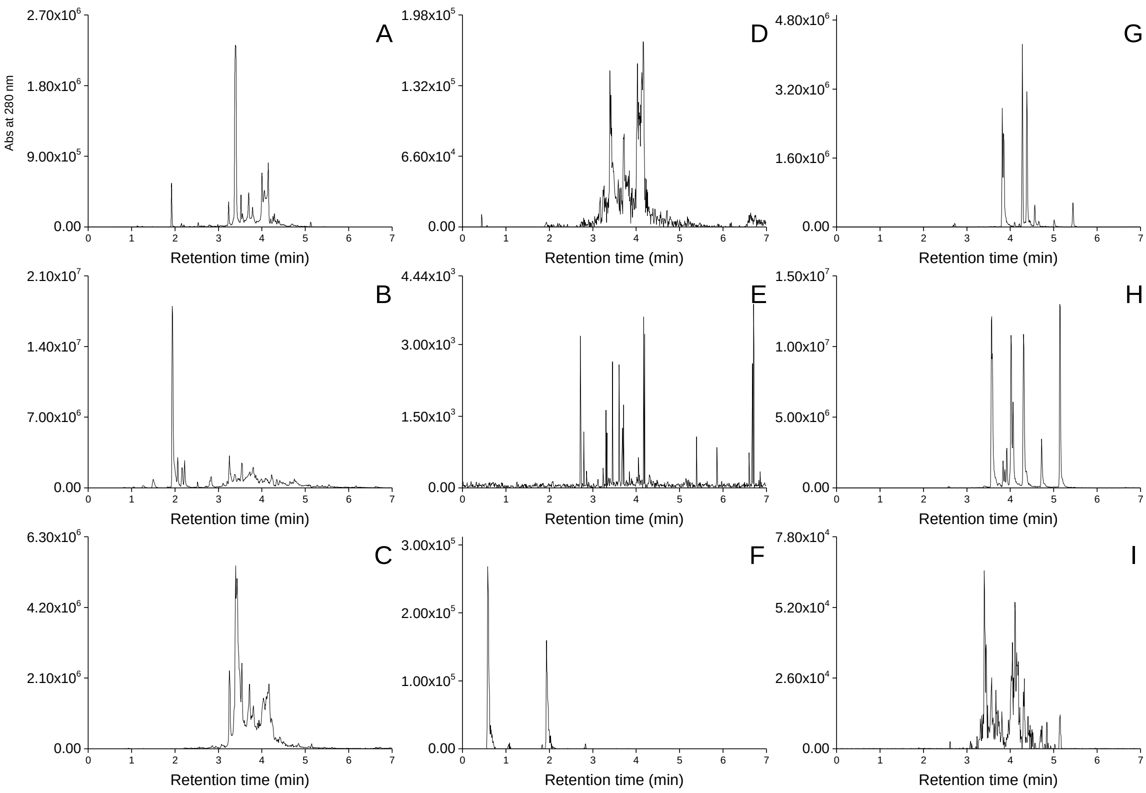

Figure S23. Examples of UHPLC-UV and group-specific UHPLC-MS/MS fingerprints recorded from the polyphenol extract of *Geranium pratense*. (A) UV traces at 280 nm, (B) galloyl derivative fingerprint, (C) hexahydroxydiphenoyl derivative fingerprint, (D) procyanidin polymer fingerprint, (E) prodelphinidin polymer fingerprint, (F) quinic acid derivative fingerprint (the peak in at 0.8 min is free quinic acid found in plants, i.e. it is not a polyphenol), (G) kaempferol derivative fingerprint, (H) quercetin derivative fingerprint and (I) myricetin derivative fingerprint. The y-axes are scaled to the most intensive peak of each fingerprint.

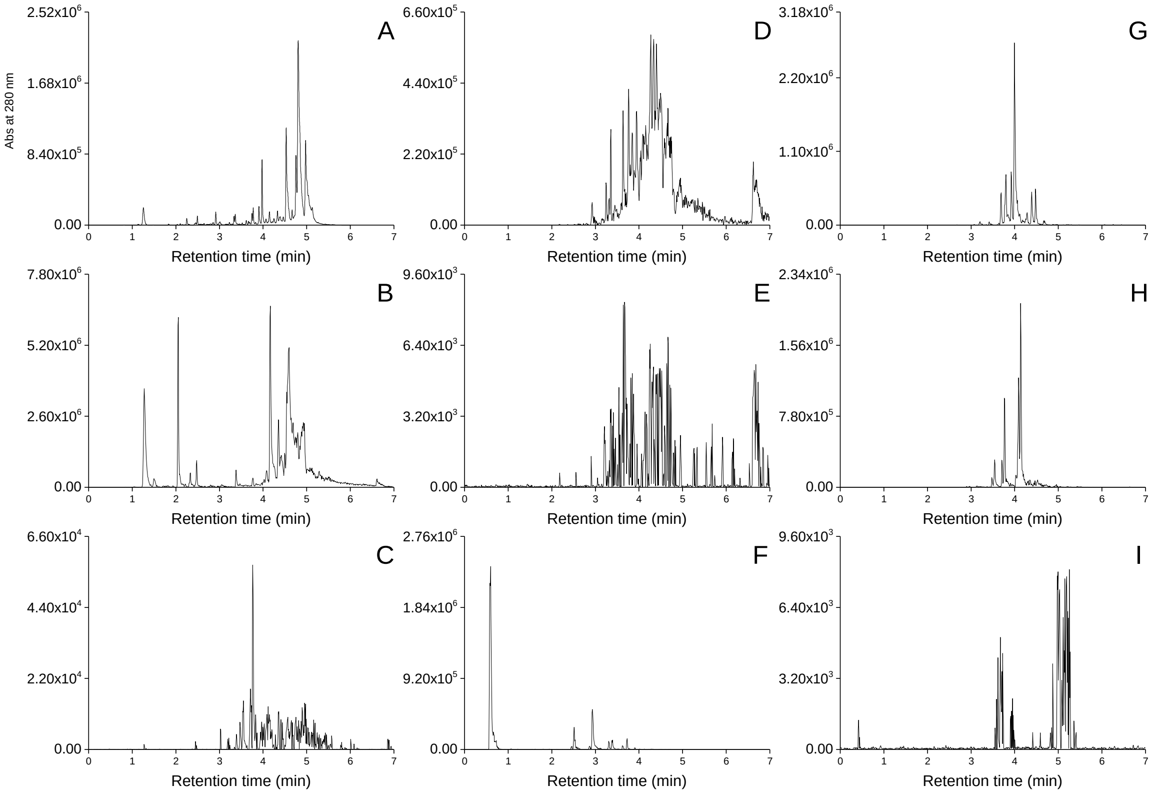

Figure S24. Examples of UHPLC-UV and group-specific UHPLC-MS/MS fingerprints recorded from the polyphenol extract of *Acer platanoides*. (A) UV traces at 280 nm, (B) galloyl derivative fingerprint, (C) hexahydroxydiphenoyl derivative fingerprint, (D) procyanidin polymer fingerprint, (E) prodelphinidin polymer fingerprint, (F) quinic acid derivative fingerprint (the peak in at 0.8 min is free quinic acid found in plants, i.e. it is not a polyphenol), (G) kaempferol derivative fingerprint, (H) quercetin derivative fingerprint and (I) myricetin derivative fingerprint. The y-axes are scaled to the most intensive peak of each fingerprint.

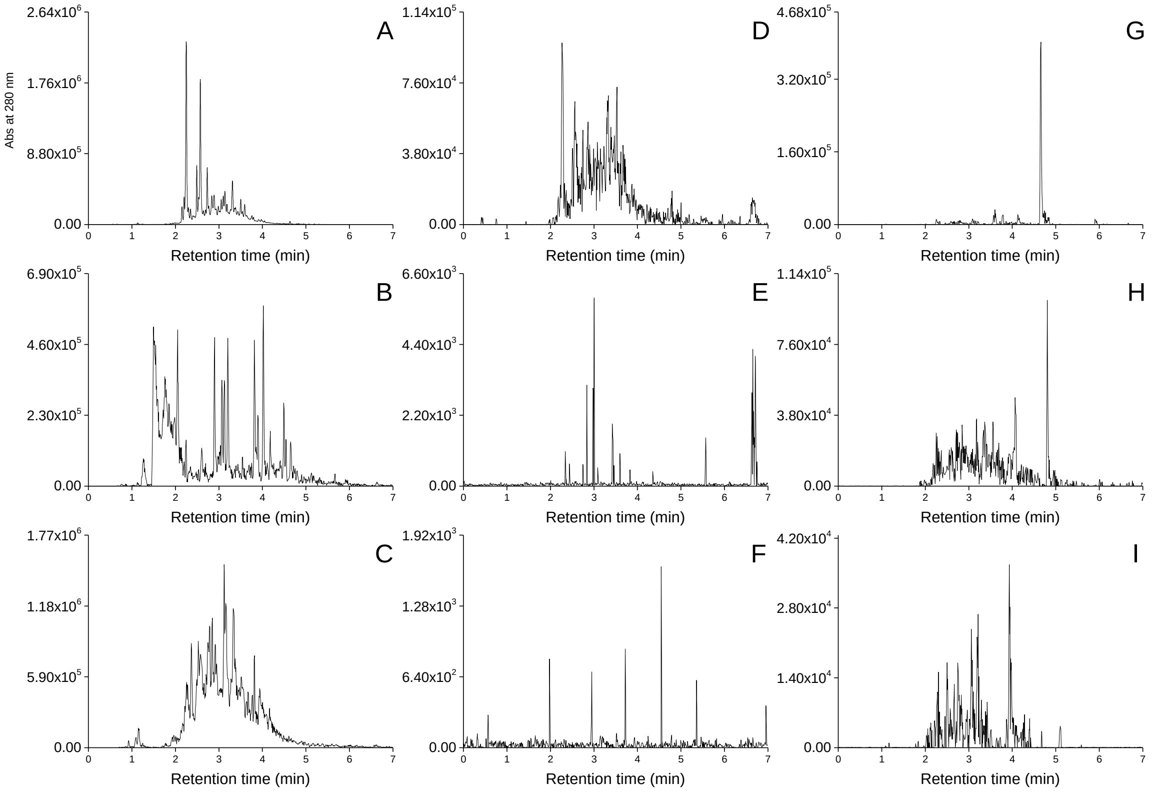

Figure S25. Examples of UHPLC-UV and group-specific UHPLC-MS/MS fingerprints recorded from the polyphenol extract of *Lythrum salicaria*. (A) UV traces at 280 nm, (B) galloyl derivative fingerprint, (C) hexahydroxydiphenoyl derivative fingerprint, (D) procyanidin polymer fingerprint, (E) prodelphinidin polymer fingerprint, (F) quinic acid derivative fingerprint (the peak in at 0.8 min is free quinic acid found in plants, i.e. it is not a polyphenol), (G) kaempferol derivative fingerprint, (H) quercetin derivative fingerprint and (I) myricetin derivative fingerprint. The y-axes are scaled to the most intensive peak of each fingerprint.

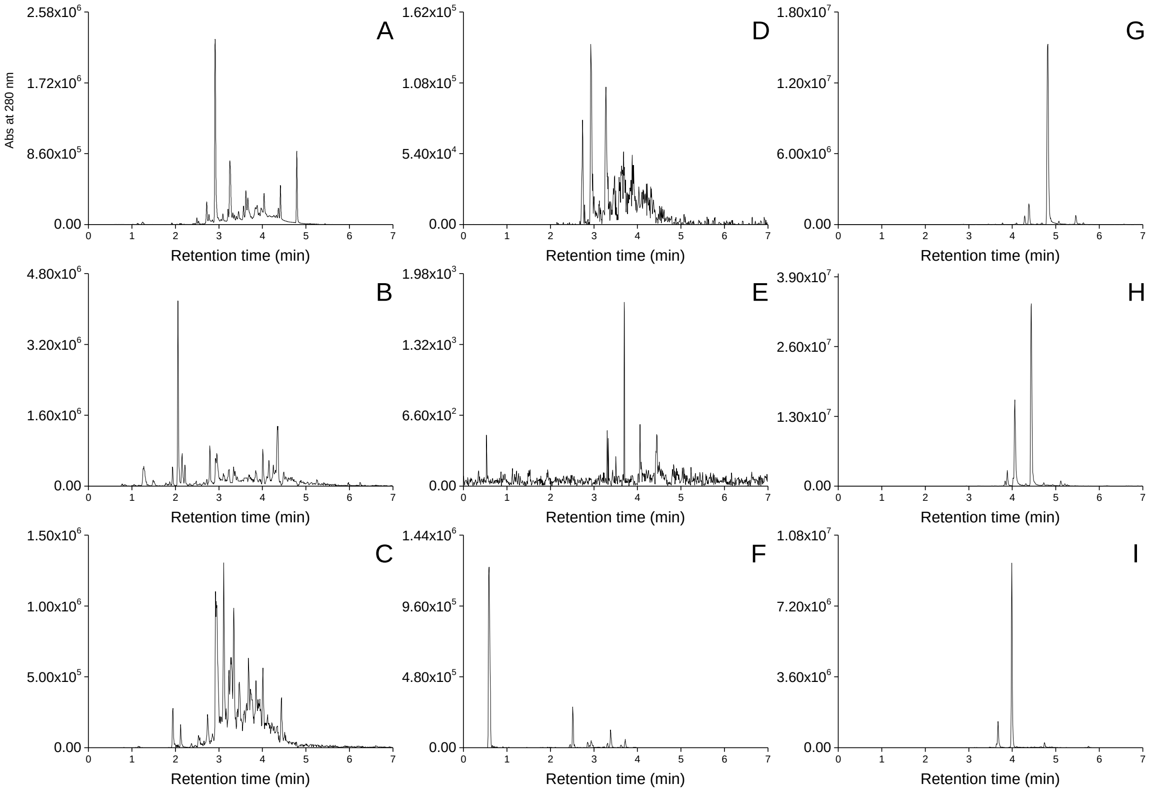

Figure S26. Examples of UHPLC-UV and group-specific UHPLC-MS/MS fingerprints recorded from the polyphenol extract of *Chamaenerion angustifolium*. (A) UV traces at 280 nm, (B) galloyl derivative fingerprint, (C) hexahydroxydiphenoyl derivative fingerprint, (D) procyanidin polymer fingerprint, (E) prodelphinidin polymer fingerprint, (F) quinic acid derivative fingerprint (the peak in at 0.8 min is free quinic acid found in plants, i.e. it is not a polyphenol), (G) kaempferol derivative fingerprint, (H) quercetin derivative fingerprint and (I) myricetin derivative fingerprint. The y-axes are scaled to the most intensive peak of each fingerprint.

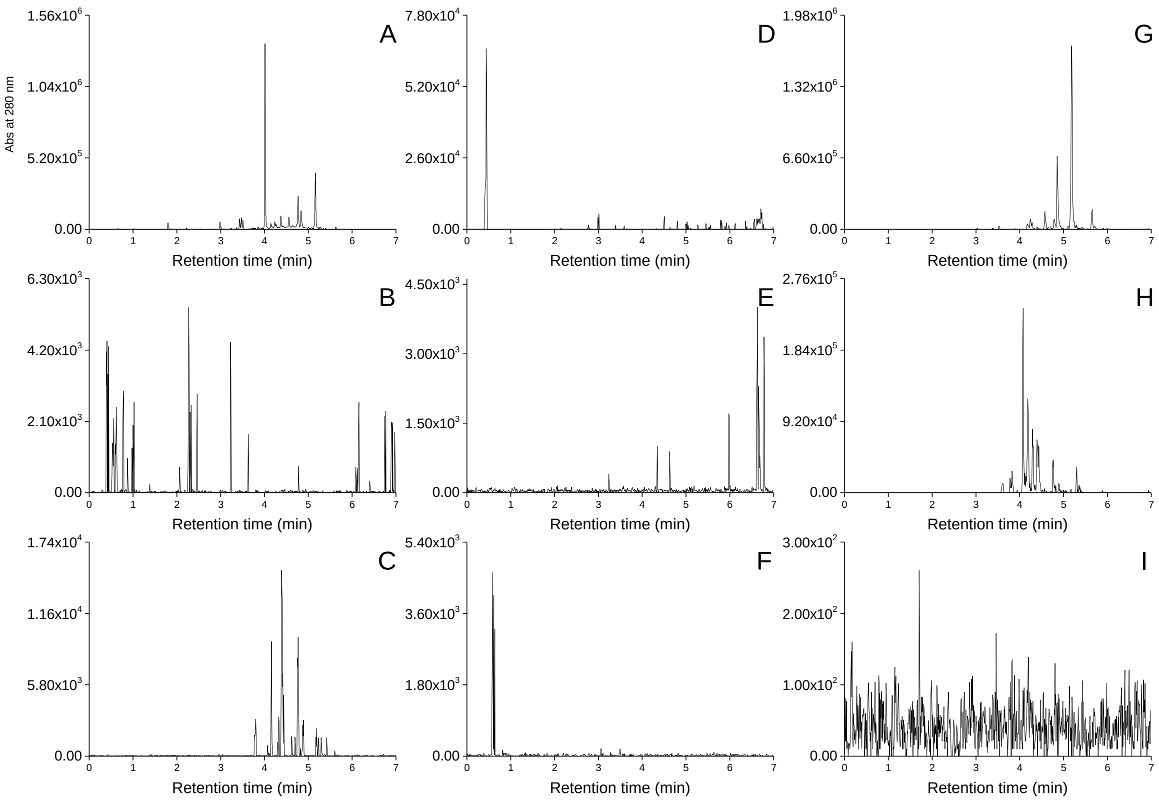

Figure S27. Examples of UHPLC-UV and group-specific UHPLC-MS/MS fingerprints recorded from the polyphenol extract of *Plantago major*. (A) UV traces at 280 nm, (B) galloyl derivative fingerprint, (C) hexahydroxydiphenoyl derivative fingerprint, (D) procyanidin polymer fingerprint, (E) prodelphinidin polymer fingerprint, (F) quinic acid derivative fingerprint (the peak in at 0.8 min is free quinic acid found in plants, i.e. it is not a polyphenol), (G) kaempferol derivative fingerprint, (H) quercetin derivative fingerprint and (I) myricetin derivative fingerprint. The y-axes are scaled to the most intensive peak of each fingerprint.

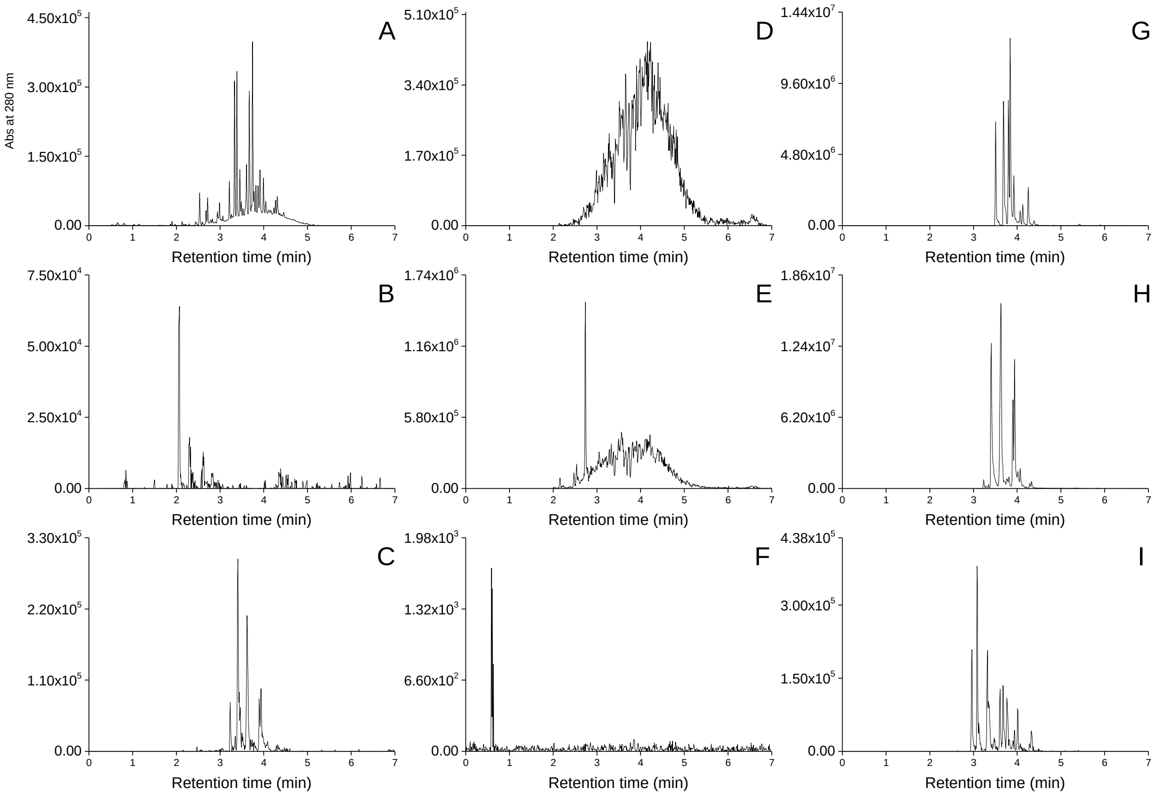

Figure S28. Examples of UHPLC-UV and group-specific UHPLC-MS/MS fingerprints recorded from the polyphenol extract of *Primula veris*. (A) UV traces at 280 nm, (B) galloyl derivative fingerprint, (C) hexahydroxydiphenoyl derivative fingerprint, (D) procyanidin polymer fingerprint, (E) prodelphinidin polymer fingerprint, (F) quinic acid derivative fingerprint (the peak in at 0.8 min is free quinic acid found in plants, i.e. it is not a polyphenol), (G) kaempferol derivative fingerprint, (H) quercetin derivative fingerprint and (I) myricetin derivative fingerprint. The y-axes are scaled to the most intensive peak of each fingerprint.

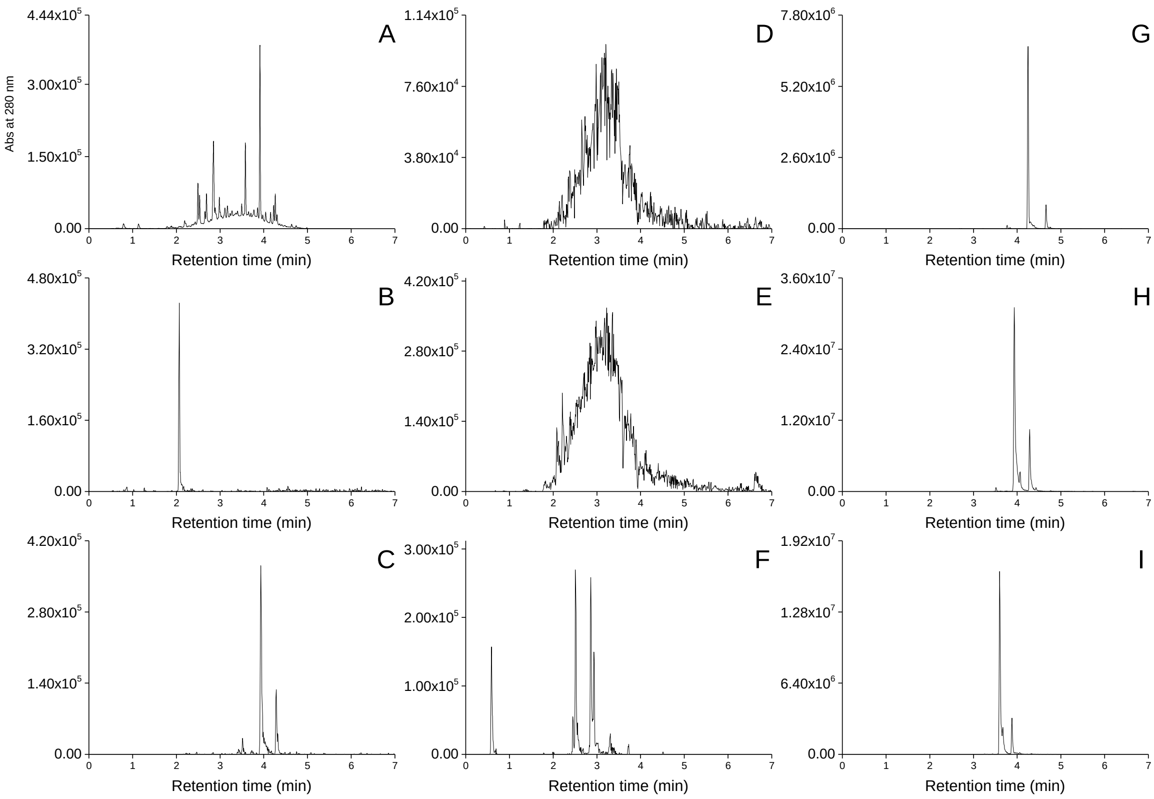

Figure S29. Examples of UHPLC-UV and group-specific UHPLC-MS/MS fingerprints recorded from the polyphenol extract of *Lysimachia vulgaris*. (A) UV traces at 280 nm, (B) galloyl derivative fingerprint, (C) hexahydroxydiphenoyl derivative fingerprint, (D) procyanidin polymer fingerprint, (E) prodelphinidin polymer fingerprint, (F) quinic acid derivative fingerprint (the peak in at 0.8 min is free quinic acid found in plants, i.e. it is not a polyphenol), (G) kaempferol derivative fingerprint, (H) quercetin derivative fingerprint and (I) myricetin derivative fingerprint. The y-axes are scaled to the most intensive peak of each fingerprint.

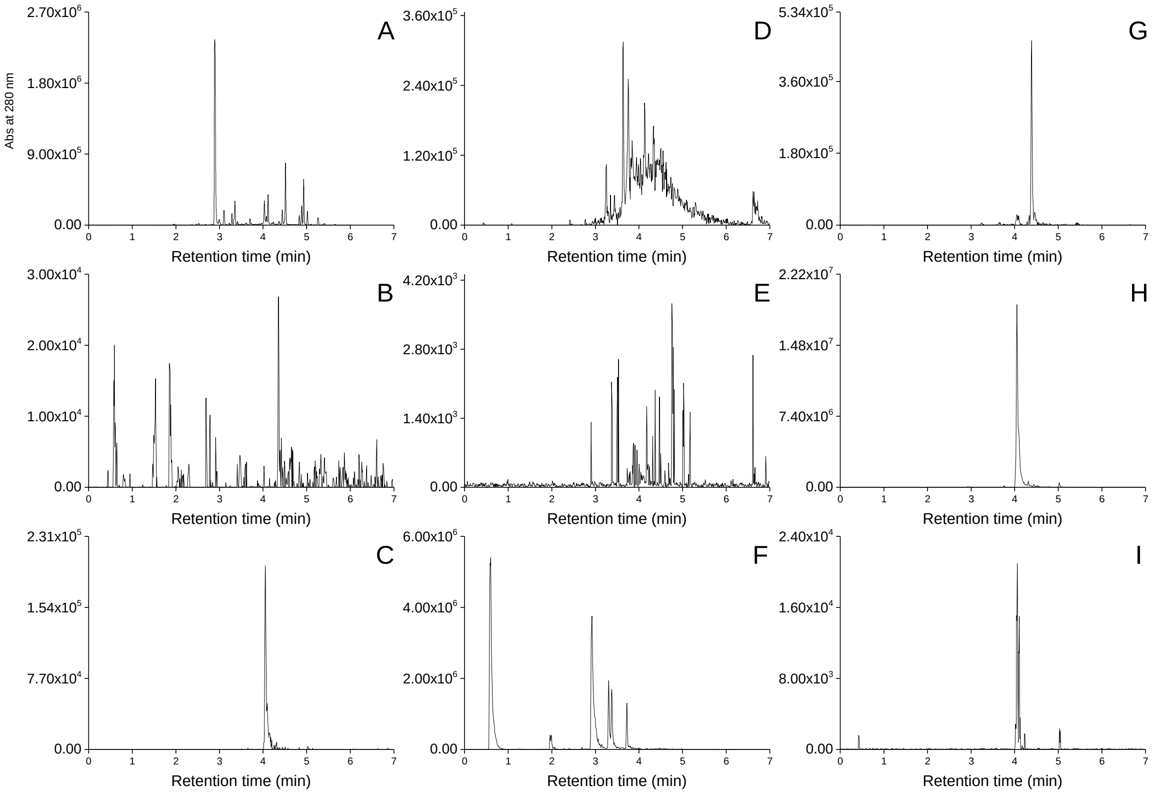

Figure S30. Examples of UHPLC-UV and group-specific UHPLC-MS/MS fingerprints recorded from the polyphenol extract of *Vaccinium myrtillus*. (A) UV traces at 280 nm, (B) galloyl derivative fingerprint, (C) hexahydroxydiphenoyl derivative fingerprint, (D) procyanidin polymer fingerprint, (E) prodelphinidin polymer fingerprint, (F) quinic acid derivative fingerprint (the peak in at 0.8 min is free quinic acid found in plants, i.e. it is not a polyphenol), (G) kaempferol derivative fingerprint, (H) quercetin derivative fingerprint and (I) myricetin derivative fingerprint. The y-axes are scaled to the most intensive peak of each fingerprint.

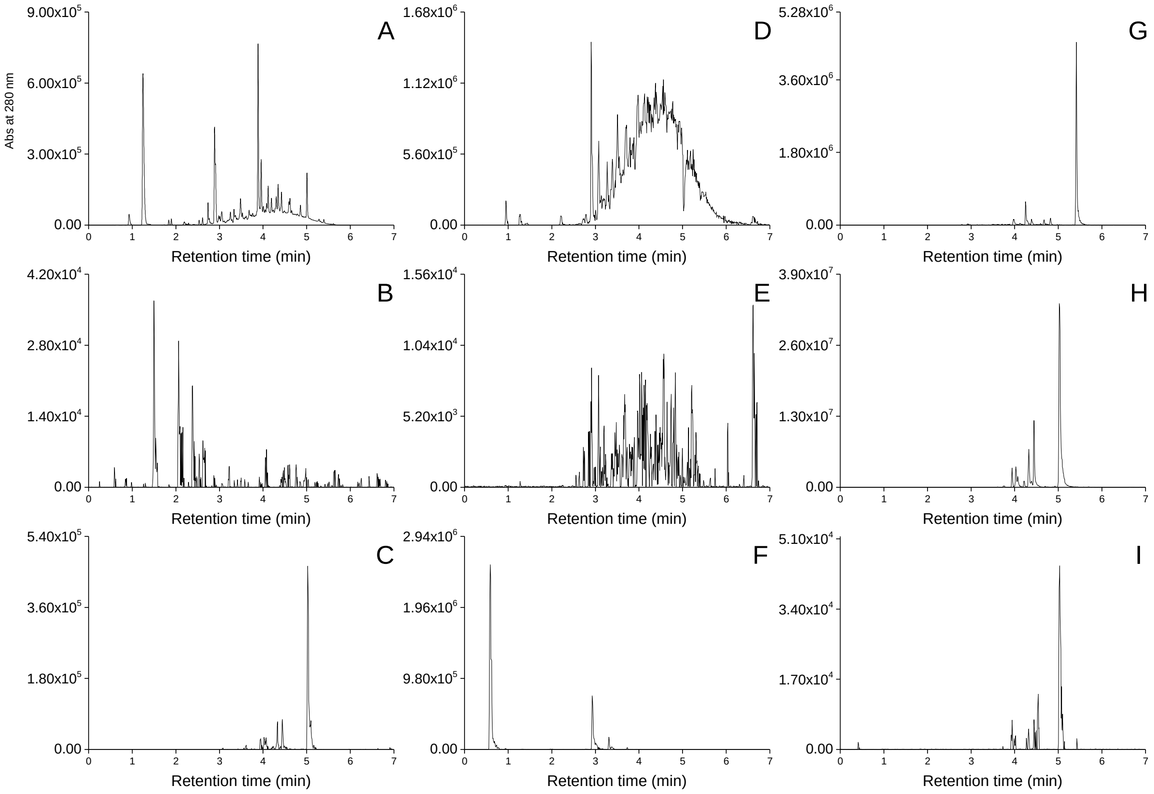

Figure S31. Examples of UHPLC-UV and group-specific UHPLC-MS/MS fingerprints recorded from the polyphenol extract of *Vaccinium vitis-idaea*. (A) UV traces at 280 nm, (B) galloyl derivative fingerprint, (C) hexahydroxydiphenoyl derivative fingerprint, (D) procyanidin polymer fingerprint, (E) prodelphinidin polymer fingerprint, (F) quinic acid derivative fingerprint (the peak in at 0.8 min is free quinic acid found in plants, i.e. it is not a polyphenol), (G) kaempferol derivative fingerprint, (H) quercetin derivative fingerprint and (I) myricetin derivative fingerprint. The y-axes are scaled to the most intensive peak of each fingerprint.

Plant evolution and sample details

Our data included 31 biologically variable plant species from evolutionary distant to closely related species. Species phylogenetic tree in relative time scale is presented in Figure S32. The tree was constructed with Phylomatic web service (version 3 in 2021, Webb & Donoghue 2005) using Zanne2014 megatree as a backbone for divergence times (Zanne et al. 2014)**.** The tree was visualized with iTOL (Interactive Tree of Life) web service (Letunic & Bork 2021).

Collection dates, coordinates, species' growth forms and collected plant parts are described in Table S2.

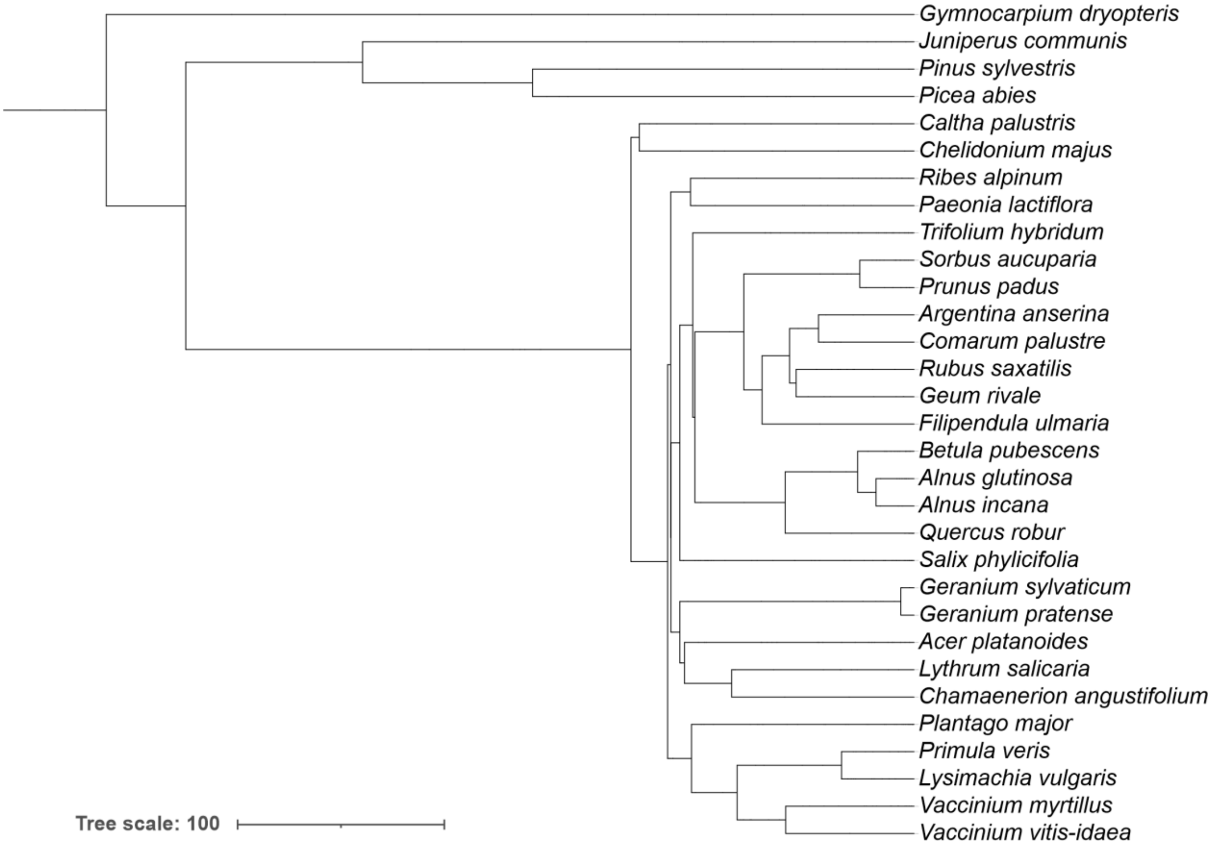

Figure S32. Phylogenetic tree of 31 plant species in relative time scale.

Table S1. The initial concentrations (mg/g, dry weight) of the polyphenol classes and bioactivities of the studied plant populations. The concentration values are population averages ± standard error of the mean. ND: not detected, NS: not significant.

	Ferns	Gymnosperms			Angiosperms				
	Cystopteridaceae	Cupressaceae	Pinaceae		Ranunculaceae	Papaveraceae	Grossulariaceae	Paeoniaceae	Fabaceae
**Class**	*Gymnocarpium dryopteris*	*Juniperus communis*	*Pinus sylvestris*	*Picea abies*	*Caltha palustris*	*Chelidonium majus*	*Ribes alpinium*	*Paeonia lactiflora*	*Trifolium hybridum*
Gallic acid derivatives	ND	ND	ND	ND	ND	ND	ND	13.7 ± 0.9	ND
HHDP derivatives	ND	ND	ND	ND	ND	ND	ND	ND	ND
**Total hydrolysable tannins**	ND	ND	ND	ND	ND	ND	ND	13.7 ± 0.9	ND
Procyanidins	8.1 ± 0.4	32.3 ± 2.4	6.0 ± 0.6	29.0 ± 3.4	ND	ND	2.6 ± 0.2	ND	40.2 ± 2.3
Prodelphinidins	10.0 ± 0.5	3.5 ± 0.8	14.1 ± 0.7	7.1 ± 1.5	ND	ND	57.4 ± 2.5	ND	ND
**Total proanthocyanidins**	18.1 ± 0.8	35.9 ± 1.8	20.1 ± 1	36.1 ± 2.3	ND	ND	60.0 ± 2.6	ND	40.2 ± 2.3
Mean degree of polymerization	14.5 ± 0.4	7.6 ± 0.3	7.9 ± 0.2	8.9 ± 0.5	-	-	12.2 ± 0.1	-	6.6 ± 0.1
Kaempferol derivatives	4.7 ± 0.2	1.0 ± 0.1	0.6 ± 0.1	5.2 ± 0.2	0.2 ± 0.1	NS	0.6 ± 0.1	3.5 ± 0.4	1.3 ± 0.1
Quercetin derivatives	0.6 ± 0.0	3.7 ± 0.2	1.1 ± 0.1	1.5 ± 0.1	2.3 ± 0.3	0.4 ± 0.1	1.3 ± 0.1	7.0 ± 0.4	6.7 ± 0.2
Myricetin derivatives	NS	0.2 ± 0.0	0.3 ± 0.0	0.5 ± 0.1	ND	ND	8.1 ± 0.3	0.1 ± 0.0	ND
**Total flavonol derivatives**	5.3 ± 0.2	4.9 ± 0.2	2.0 ± 0.2	7.2 ± 0.2	2.5 ± 0.4	0.4 ± 0.1	9.9 ± 0.4	10.6 ± 0.5	8.0 ± 0.2
**Quinic acid derivatives**	8.9 ± 0.4	0.8 ± 0.1	ND	0.9 ± 0.0	ND	ND	0.9 ± 0.1	ND	ND
Total sum of detected polyphenols	32.3 ± 1.0	41.6 ± 1.9	22.2 ± 1.1	44.1 ± 2.3	2.5 ± 0.4	0.4 ± 0.1	71.0 ± 2.4	24.4 ± 0.8	48.7 ± 2.4
Total phenolics	42.7 ± 2.3	42.2 ± 1.3	32.1 ± 1.1	43.6 ± 2.4	18.4 ± 1.9	5.2 ± 0.4	61.7 ± 2.2	104.4 ± 8.3	57.7 ± 2.4
Oxidative activity	15.6 ± 1.4	5.3 ± 0.9	8.8 ± 0.4	5.2 ± 1.2	5.5 ± 0.8	1.4 ± 0.2	26.5 ± 1.1	0.4 ± 0.2	0.7 ± 0.2
Protein precipitation capacity	2.3 ± 0.5	7.3 ± 1.3	0.3 ± 0.1	10.0 ± 1.8	ND	ND	14.9 ± 1.7	72.2 ± 9.1	12.7 ± 1.5

Table S1. Continues. The initial concentrations (mg/g, dry weight) of the polyphenol classes and bioactivities of the studied plant populations. The concentration values are population averages ± standard error of the mean. ND: not detected, NS: not significant.

	Angiosperms										
	Rosaceae							Betulaceae			Fagaceae
**Class**	*Sorbus aucuparia*	*Prunus padus*	*Argentina anserina*	*Comarum palustre*	*Rubus saxatilis*	*Geum rivale*	*Filipendula ulmaria*	*Betula pubescens*	*Alnus glutinosa*	*Alnus incana*	*Quercus robur*
Gallic acid derivatives	ND	ND	1.3 ± 0.1	6.6 ± 0.9	2.2 ± 0.4	1.9 ± 0.1	9.7 ± 0.5	4.6 ± 0.4	1.2 ± 0.5	ND	5.6 ± 0.4
HHDP derivatives	ND	ND	27.7 ± 1.0	53.9 ± 2.0	34.5 ± 1.4	44.6 ± 1.3	17.0 ± 0.4	16.5 ± 2.0	3.4 ± 2.5	ND	33.7 ± 1.6
**Total hydrolysable tannins**	ND	ND	29.0 ± 1.0	60.5 ± 2.7	36.7 ± 1.8	46.5 ± 1.3	26.8 ± 0.9	21.1 ± 2.3	4.6 ± 3.0	ND	39.2 ± 2.0
Procyanidins	26.8 ± 2.8	12.9 ± 2.2	7.3 ± 0.8	0.9 ± 0.3	0.5 ± 0.2	6.1 ± 0.7	19.7 ± 2.2	7.8 ± 0.4	0.6 ± 0.3	0.3 ± 0.2	1.0 ± 0.5
Prodelphinidins	ND	ND	6.9 ± 0.7	ND	ND	ND	ND	15.7 ± 1.5	ND	ND	NS
**Total proanthocyanidins**	27.0 ± 2.8	13.0 ± 2.3	14.2 ± 1.5	0.9 ± 0.3	0.5 ± 0.2	6.1 ± 0.7	19.8 ± 2.3	23.5 ± 1.8	0.6 ± 0.3	0.3 ± 0.2	1.0 ± 0.5
Mean degree of polymerization	6.4 ± 0.1	4.2 ± 0.2	6.1 ± 0.2	2.8 ± 0.2	1.6 ± 0.1	2.3 ± 0.1	2.1 ± 0.1	10.2 ± 0.3	2.0 ± 0.1	2.1 ± 0.2	2.1 ± 0.3
Kaempferol derivatives	4.5 ± 0.6	0.8 ± 0.1	2.0 ± 0.1	2.3 ± 0.3	1.3 ± 0.1	0.4 ± 0.0	1.1 ± 0.1	4.4 ± 0.3	0.3 ± 0.0	0.4 ± 0.0	9.0 ± 0.4
Quercetin derivatives	7.2 ± 0.2	6.4 ± 0.3	3.7 ± 0.1	2.7 ± 0.2	2.2 ± 0.2	0.7 ± 0.1	9.1 ± 0.6	5.8 ± 0.2	2.2 ± 0.1	5.1 ± 0.2	3.5 ± 0.3
Myricetin derivatives	0.2 ± 0.0	ND	2.4 ± 0.1	ND	ND	ND	ND	0.6 ± 0.1	ND	ND	0.1 ± 0.0
**Total flavonol derivatives**	11.8 ± 0.7	7.2 ± 0.3	8.2 ± 0.2	5.0 ± 0.3	3.5 ± 0.2	1.2 ± 0.1	10.1 ± 0.8	10.7 ± 0.4	2.5 ± 0.1	5.5 ± 0.2	12.5 ± 0.4
**Quinic acid derivatives**	13.3 ± 0.6	6.8 ± 0.4	ND	ND	ND	2.7 ± 0.2	1.4 ± 0.6	7.7 ± 0.6	3.3 ± 0.4	4.7 ± 0.3	1.0 ± 0.2
Total sum of detected polyphenols	52.2 ± 3.2	27.1 ± 2.8	51.3 ± 1.5	66.8 ± 2.6	40.9 ± 1.6	56.4 ± 1.6	58.1 ± 2.7	63 ± 2.1	11 ± 3.5	11.1 ± 0.4	53.8 ± 1.9
Total phenolics	54.8 ± 3.0	30.6 ± 2.9	75.7 ± 1.9	95.3 ± 3.1	62.2 ± 2.2	75.0 ± 2.8	86.7 ± 3.1	61.1 ± 1.5	46.4 ± 4.4	43.1 ± 2.7	89.2 ± 2.7
Oxidative activity	8.8 ± 0.8	2.2 ± 0.5	22.7 ± 0.6	30.3 ± 1.1	19.6 ± 0.8	22.6 ± 0.8	15.1 ± 0.7	18.8 ± 0.7	21.1 ± 2.0	16.6 ± 1.4	37.4 ± 1.3
Protein precipitation capacity	7.5 ± 2.3	1.0 ± 1.1	43.6 ± 2.7	67.1 ± 3.4	29.0 ± 2.5	38.1 ± 2.6	21.4 ± 2.0	16.1 ± 1.4	2.5 ± 2.3	ND	37.6 ± 4.9

Table S1. Continues. The initial concentrations (mg/g, dry weight) of the polyphenol classes and bioactivities of the studied plant populations. The concentration values are population averages ± standard error of the mean. ND: not detected, NS: not significant.

	Angiosperms										
	Salicaceae	Geraniaceae		Sapindaceae	Lythraceae	Onagraceae	Plantaginaceae	Primulaceae		Ericaceae	
**Class**	*Salix phylicifolia*	*Greanium sylvaticum*	*Geranium pratense*	*Acer platanoides*	*Lythrum salicaria*	*Chamaenerion angustifolium*	*Plantago major*	*Primula veris*	*Lysimachia vulgaris*	*Vaccinium myrtillus*	*Vaccinium vitis-idaea*
Gallic acid derivatives	ND	24.1 ± 0.8	24.5 ± 0.7	18.3 ± 1.3	2.1 ± 0.2	8.2 ± 0.2	ND	ND	ND	ND	ND
HHDP derivatives	ND	60.9 ± 1.4	58.7 ± 1.3	ND	28.0 ± 1.2	24.5 ± 0.5	ND	ND	ND	ND	ND
**Total hydrolysable tannins**	ND	85.0 ± 1.5	83.2 ± 1.9	18.3 ± 1.3	30.1 ± 1.4	32.7 ± 0.6	ND	ND	ND	ND	ND
Procyanidins	5.9 ± 1.4	2.1 ± 0.1	ND	8.1 ± 1.5	ND	ND	ND	9.6 ± 0.4	1.9 ± 0.1	6.3 ± 0.6	64.1 ± 1.8
Prodelphinidins	23.4 ± 3.5	0.8 ± 0.1	ND	NS	ND	ND	ND	27.9 ± 1.3	21.9 ± 1.2	ND	NS
**Total proanthocyanidins**	29.3 ± 4.7	2.9 ± 0.1	ND	8.1 ± 1.5	ND	ND	ND	37.5 ± 1.5	23.7 ± 1.2	6.3 ± 0.6	64.7 ± 1.8
Mean degree of polymerization	16.6 ± 0.8	2.1 ± 0.1	-	3.4 ± 0.1	-	-	-	7.2 ± 0.1	16.8 ± 0.2	3.8 ± 0.2	4.2 ± 0.1
Kaempferol derivatives	0.5 ± 0.1	1.7 ± 0.2	2.7 ± 0.2	1.7 ± 0.1	0.1 ± 0.0	7.4 ± 0.2	0.8 ± 0.0	4.9 ± 0.3	2.9 ± 0.3	0.2 ± 0.0	1.1 ± 0.1
Quercetin derivatives	1.9 ± 0.2	5.0 ± 0.2	8.8 ± 0.3	0.6 ± 0.1	NS	7.8 ± 0.1	ND	4.5 ± 0.2	4.7 ± 0.2	3.4 ± 0.2	6.9 ± 0.2
Myricetin derivatives	2.9 ± 0.4	0.2 ± 0.0	NS	ND	ND	1.9 ± 0.0	ND	0.4 ± 0.0	4.1 ± 0.2	ND	ND
**Total flavonol derivatives**	5.4 ± 0.5	6.9 ± 0.3	11.6 ± 0.3	2.3 ± 0.2	0.1 ± 0.0	17.1 ± 0.1	0.8 ± 0.0	9.8 ± 0.4	11.7 ± 0.5	3.6 ± 0.2	8.0 ± 0.2
**Quinic acid derivatives**	1.5 ± 0.6	8.4 ± 0.2	0.5 ± 0.0	1.4 ± 0.3	ND	1.5 ± 0.0	ND	ND	1.3 ± 0.1	25.6 ± 1.1	2.9 ± 0.2
Total sum of detected polyphenols	36.5 ± 4.7	103.3 ± 1.2	95.5 ± 1.8	30.6 ± 2.6	30.4 ± 1.4	51.2 ± 0.6	0.9 ± 0.0	48 ± 1.6	37 ± 1.6	35.5 ± 1.4	75.7 ± 1.7
Total phenolics	83.8 ± 2.5	146.7 ± 5.3	142.3 ± 7.3	86.8 ± 6.5	146.5 ± 13.4	152.1 ± 4.3	27.3 ± 1.0	65 ± 1.5	76.4 ± 4.3	54.6 ± 1.7	103.1 ± 0.7
Oxidative activity	27.0 ± 1.1	36.3 ± 1.3	32.1 ± 1.1	1.3 ± 0.8	65.3 ± 6.6	35.8 ± 1.7	9.5 ± 0.3	21.2 ± 1.0	34.2 ± 2.1	16.9 ± 0.8	15.1 ± 0.6
Protein precipitation capacity	10.9 ± 3.4	56.9 ± 3.0	62.0 ± 4.6	53.5 ± 8.3	94.2 ± 9.0	73.7 ± 2.7	ND	9.1 ± 1.4	10.3 ± 1.4	0.3 ± 0.3	23.8 ± 1.8

Table S2. Collected plant species, their growth forms, collected organs, collection dates and coordinates. The species are listed in a phylogenetic order presented in Figure S32.

Species	Growth form	Organ	Date	Coordinates	
Gymnocarpium dryopteris	perennial fern	leaves	3.6.2016	N 60° 27.396'	E 22° 22.558'
Juniperus communis	coniferous tree	new growth needles	31.5.2016	N 60° 27.505'	E 22° 18.773'
Pinus sylvestris	coniferous tree	old needles	26.7.2016	N 60° 27.606'	E 22° 18.967'
Picea abies	coniferous tree	new growth needles	27.5.2016	N 60° 28.060'	E 22° 19.739'
Caltha palustris	perennial herbaceous	leaves	7.6.2016	N 60° 27.888'	E 22° 19.407'
Chelidonium majus	perennial herbaceous	leaves	6.7.2016	N 60° 27.710'	E 22° 16.921'
Ribes alpinum	deciduous shrub	leaves	20.7.2016	N 60° 27.251'	E 22° 20.065'
Paeonia lactiflora	perennial herbaceous	leaves	29.6.2016	N 60° 26.041'	E 22° 10.322'
Trifolium hybridum	perennial herbaceous	flowers	1.7.2016	N 60° 27.443'	E 22° 17.243'
Sorbus aucuparia	deciduous tree	leaves	31.5.2016	N 60° 27.512'	E 22° 18.659'
Prunus padus	deciduous tree	leaves	2.6.2016	N 60° 27.710'	E 22° 16.921'
Argentina anserina	perennial herbaceous	leaves	5.7.2016	N 60° 27.475'	E 22° 17.245'
Comarum palustre	perennial herbaceous	leaves	7.7.2016	N 60° 27.532'	E 22° 22.523'
Rubus saxatilis	perennial herbaceous	leaves	13.6.2016	N 60° 27.251'	E 22° 20.065'
Geum rivale	perennial herbaceous	leaves	20.7.2016	N 60° 27.623'	E 22° 18.901'
Filipendula ulmaria	perennial herbaceous	leaves	30.6.2016	N 60° 27.888'	E 22° 19.407'
Betula pubescens	deciduous tree	leaves	25.5.2016	N 60° 26.083'	E 22° 10.397'
Alnus glutinosa	deciduous tree	leaves	30.5.2016	N 60° 26.238'	E 22° 12.247'
Alnus incana	deciduous tree	leaves	27.5.2016	N 60° 27.872'	E 22° 19.274'
Quercus robur	deciduous tree	leaves	30.5.2016	N 60° 26.200'	E 22° 10.280'
Salix phylicifolia	deciduous tree	leaves	3.6.2016	N 60° 27.576'	E 22° 22.473'
Geranium sylvaticum	perennial herbaceous	leaves	20.7.2016	N 60° 27.688'	E 22° 19.186'
Geranium pratense	perennial herbaceous	leaves	29.6.2016	N 60° 26.151'	E 22° 10.511'
Acer platanoides	deciduous tree	leaves	2.6.2016	N 60° 27.444'	E 22° 16.679'
Lythrum salicaria	perennial herbaceous	leaves	5.7.2016	N 60° 26.567'	E 22° 12.529'
Chamaenerion angustifolium	perennial herbaceous	flowers	1.7.2016	N 60° 27.475'	E 22° 17.245'
Plantago major	perennial herbaceous	leaves	5.7.2016	N 60° 26.238'	E 22° 12.247'
Primula veris	perennial herbaceous	flowers	25.5.2016	N 60° 26.151'	E 22° 10.511'
Lysimachia vulgaris	perennial herbaceous	leaves	30.6.2016	N 60° 27.872'	E 22° 19.274'
Vaccinium myrtillus	perennial dwarf shrub	leaves	27.5.2016	N 60° 28.060'	E 22° 19.739'
Vaccinium vitis-idaea	perennial dwarf shrub	leaves	7.6.2016	N 60° 28.179'	E 22° 19.877'

Normalization limits for mass spectrometric fingerprint mapping

Table S3. The initial concentration values (mg/g) of each polyphenol group and bioactivity at normalized scale (0.0-1.0), detection limit (0.0) and detected maximal values (max).

	**0.0**	**0.2**	**0.4**	**0.6**	**0.8**	**1.0**	**max**
Gallic acid derivatives	0.1	5.0	10.0	15.0	20.0	25.0	98.6
HHDP derivatives	0.1	15.0	30.0	45.0	60.0	75.0	213.6
**Total hydrolysable tannins**	0.1	17.0	34.0	51.0	68.0	85.0	244.9
Procyanidins	1.0	15.0	30.0	45.0	60.0	75.0	307.4
Prodelphinidins	1.0	9.0	18.0	27.0	36.0	45.0	212.2
**Total proanthocyanidins**	1.0	17.0	34.0	51.0	68.0	85.0	309.0
Kaempferol derivatives	0.1	1.2	2.4	3.6	4.8	6.0	40.2
Quercetin derivatives	0.1	1.7	3.4	5.1	6.8	8.5	24.9
Myricetin derivatives	0.1	1.2	2.4	3.6	4.8	6.0	25.1
**Total flavonol derivatives**	0.1	2.5	5.0	7.5	10.0	12.5	49.2
**Quinic acid derivatives**	0.1	2.6	5.2	7.8	10.4	13.0	41.4
Oxidative activity	0.1	8.0	16.0	24.0	32.0	40.0	129.4
Protein precipitation capacity	0.1	14.0	28.0	42.0	56.0	70.0	188.4

Table S4. The initial concentration values (mg/g) of each flavonol group and glycosylation pattern at normalized scale.

	**0.0**	**0.2**	**0.4**	**0.6**	**0.8**	**1.0**	**max**
KA-3-*O*-glycosides	0.10	1.20	2.40	3.60	4.80	6.00	39.74
Other KA-glycosides	0.10	0.15	0.20	0.25	0.30	0.35	2.92
**Total kaempferol derivatives**	0.10	1.20	2.40	3.60	4.80	6.00	40.20
QU-3-*O*-glycosides	0.10	1.20	2.40	3.60	4.80	6.00	19.48
Other QU-glycosides	0.10	0.60	1.20	1.80	2.40	3.00	15.89
**Total quercetin derivatives**	0.10	1.70	3.40	5.10	6.80	8.50	24.90
MY-3-*O*-glycosides	0.10	0.80	1.60	2.40	3.20	4.00	23.11
Other MY-glycosides	0.10	0.15	0.20	0.25	0.30	0.35	2.03
**Total myricetin derivatives**	0.10	1.20	2.40	3.60	4.80	6.00	25.10
